# Membrane repair triggered by cholesterol-dependent cytolysins is activated by mixed lineage kinases and MEK

**DOI:** 10.1126/sciadv.abl6367

**Published:** 2022-03-16

**Authors:** Sucharit Ray, Robyn Roth, Peter A. Keyel

**Affiliations:** 1 Department of Biological Sciences, Texas Tech University, Lubbock, TX 79409, USA.; 2 Department of Cell Biology and Physiology, Washington University School of Medicine, St. Louis, MO 63110, USA.

## Abstract

Repair of plasma membranes damaged by bacterial pore-forming toxins, such as streptolysin O or perfringolysin O, during septic cardiomyopathy or necrotizing soft tissue infections is mediated by several protein families. However, the activation of these proteins downstream of ion influx is poorly understood. Here, we demonstrate that following membrane perforation by bacterial cholesterol-dependent cytolysins, calcium influx activates mixed lineage kinase 3 independently of protein kinase C or ceramide generation. Mixed lineage kinase 3 uncouples mitogen-activated kinase kinase (MEK) and extracellular-regulated kinase (ERK) signaling. MEK signals via an ERK-independent pathway to promote rapid annexin A2 membrane recruitment and enhance microvesicle shedding. This pathway accounted for 70% of all calcium ion-dependent repair responses to streptolysin O and perfringolysin O, but only 50% of repair to intermedilysin. We conclude that mixed lineage kinase signaling via MEK coordinates microvesicle shedding, which is critical for cellular survival against cholesterol-dependent cytolysins.

## Teaser

In contrast to previous views that calcium influx directly controls membrane repair, MEK recruits annexin A2 for repair.

## INTRODUCTION

Septic cardiomyopathy and lethal necrotizing soft tissue infections (NSTIs) can be caused by the Gram-positive bacteria *Streptococcus pyogenes* and *Clostridium perfringens*. These bacteria secrete key virulence factors in the cholesterol-dependent cytolysin (CDC) family of pore-forming toxins: streptolysin O (SLO) and perfringolysin O (PFO), respectively. SLO and PFO bind to cholesterol on the host membrane, oligomerize, and form pores in host lipid membranes (*1*). Pore formation causes rapid, reversible arrhythmia and fibrillation during septic cardiomyopathy (*2*), cell death, and immune evasion during NSTIs (*1*).Therefore, it is critical to determine the mechanisms by which CDCs are resisted by host cells.

Host cells resist lysis using a network of poorly understood membrane repair mechanisms. Currently, three main repair mechanisms protect cells. Patch repair is the fusion of vesicles with the membrane (*3*). Clogging is the assembly of a crystalline lattice to physically block the pore (*4*). Last, microvesicle shedding is the sequestration and shedding of toxins on microvesicles (*5*, *6*). A previously proposed repair mechanism, endocytosis (*7*), is now known to clear inactive toxin from the surface and restore homeostasis (*8*). Repair is triggered by toxin oligomerization and ion flux (*3*, *8*). While potassium efflux may trigger repair in response to toxins such as sticholysin and listeriolysin O (LLO) (*9*), calcium influx is generally accepted to activate all repair pathways described to date for CDCs (*1*, *3*). Calcium chelation is the current state of the art for blocking membrane repair, but it has wide reaching effects beyond membrane repair. Thus, there is a critical need to identify the signaling intermediates by which calcium activates repair pathways.

During patch repair and clogging, local Ca^2+^ influx and/or reactive oxygen species may directly activate fusion proteins and depolymerize the actin network (*3*). For example, during CDC attack, annexin membrane recruitment occurs in order of Ca^2+^ sensitivity: Annexin A2 (A2) is recruited first, then A6, and lastly A1 (*4*, *10*). However, once the cytoplasmic [Ca^2+^] exceeds the threshold level for an annexin, it is unclear how the annexin specifically targets the disruption. Annexins can be phosphorylated by kinases, such as A2 by protein kinase C (PKC) (*11*), so additional factors beyond Ca^2+^ may coordinate annexin targeting for repair.

Repair by microvesicle shedding may require signaling intermediates to recruit repair proteins that promote shedding. Annexins can induce membrane curvature, may contribute to microvesicle shedding (*12*, *13*), and are present on microvesicles shed during toxin attack (*14*, *15*). Similarly, the endosomal sorting complex required for transport (ESCRT) machinery is activated to repair membrane damage after toxin attack (*6*). During membrane repair, the ESCRT component apoptosis-linked gene interacting protein X (Alix) may be recruited by apoptosis-linked gene-2 (ALG-2) directly by Ca^2+^ influx (*16*). However, after ALG-2 binding, Alix is typically stabilized by an accessory protein, such as HIV Gag or tumor-susceptibility 101 (Tsg101) (*17*). To date, there is no role for Tsg101 in membrane repair (*6*, *18*), suggesting that another protein is needed to stabilize Alix.

There are many possible signaling pathways by which ion flux could activate membrane repair, such as mitogen-activated protein kinase (MAPK) cascades. At late time points after intoxication, rapidly accelerated fibrosarcoma kinase (RAF) is activated (*19*). RAF, p21-activated kinases (PAKs), and mixed-lineage kinases (MLKs) activate MAPK kinase (MEK) (*20*, *21*). Long-term CDC intoxication further activates stress-responsive MAPKs such as p38 and c-Jun N-terminal kinases (JNK1/2) to promote apoptosis and inflammation after unregulated K^+^ efflux (*1*). After K^+^ efflux, MEK and extracellular-regulated kinase (ERK) might promote repair (*9*), but the pathway by which this occurs is unknown. Alternatively, PKC is activated by Ca^2+^ influx and phosphorylates annexins (*11*, *22*), but the role during repair is unknown. Sphingomyelinase is activated by CDC attack (*23*, *24*). Ceramide can signal via MLK3 to activate JNK and p38 in other systems (*25*, *26*). Thus, it is unclear what signaling pathways, if any, are activated to promote repair in response to CDCs.

Since MEK/ERK is the MAPK signaling pathway associated with cell survival and proliferation, we hypothesized that MEK coordinates membrane repair. Here, we tested this hypothesis. We found that MEK activity accounts for 70% of the repair response to PFO and SLO, but only 50% of the repair response to another CDC, intermedilysin (ILY), which binds human CD59 instead of cholesterol. We found that Ca^2+^ activated MLK3, which promoted MEK-dependent, but ERK-independent, membrane repair. MEK-dependent repair did not require K^+^ efflux, ceramide, A1, or A6. MEK was necessary for the rapid recruitment of A2 to the plasma membrane and accounted for 50% of the microvesicle shedding triggered by CDCs. Overall, these results reveal a previously unidentified signaling mechanism that protects cells from CDC attack during NSTIs and septic cardiomyopathy.

## RESULTS

### MEK prevents membrane damage during septic cardiomyopathy

**Fig. 1.**
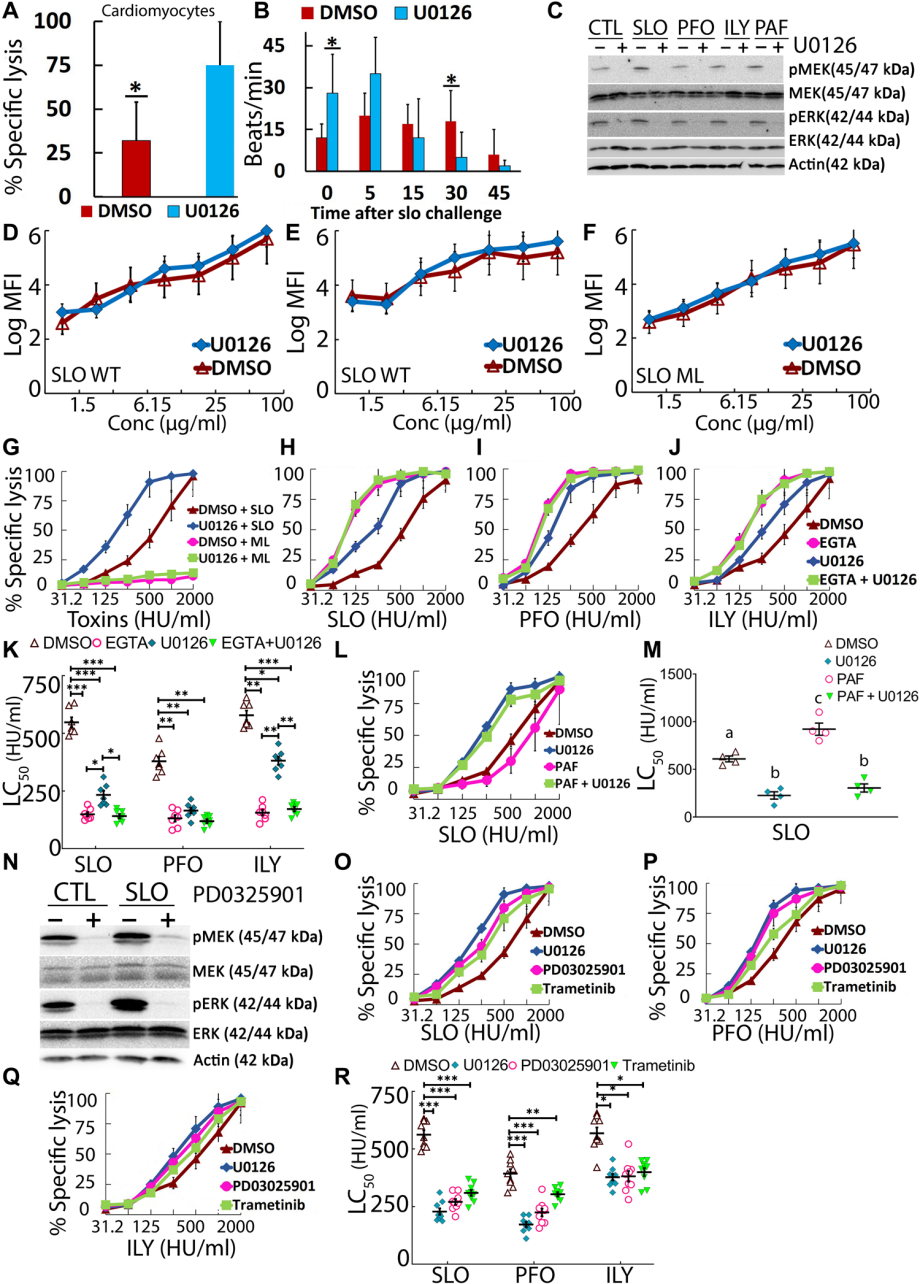
MEK protects multiple cell types from CDC-induced cell lysis. (**A** and **B**) Cardiomyocytes were pretreated with 20 μM MEK inhibitor U0126 or vehicle dimethyl sulfoxide (DMSO) for 30 min and challenged with 62.5 hemolytic units (HU)/ml SLO for 15 min at 37°C. (A) Viability was assayed by MTT [3-(4,5-dimethylthiazol-2-yl)-2,5-diphenyltetrazolium bromide] assay. (B) Beating was measured by confocal imaging. (**C** to **R**) HeLa cells were pretreated with DMSO or 20 μM inhibitors for 30 min, then challenged with toxin, and analyzed by Western blot or flow cytometry. In (C) and (N), cells were challenged with sublytic SLO, PFO, or ILY or 20 μM PAF16 for 30 min at 37°C and analyzed by Western blot. MFI, Median fluorescent intensity. (D to F) Cells were challenged with (D and E) Cy5-labeled SLO wild-type (WT) or (F) Cy5-labeled monomer-locked SLO (SLO ML) at the indicated concentrations. In (G) to (M) and (O) to (R), cells were challenged with the indicated toxins at 31 to 2000 HU/ml or 20 μM PAF16 (L and M) for 30 min at 37°C. Propidium iodide (PI) uptake and toxin binding were analyzed by flow cytometry. In (H) to (K), 2 mM EGTA was used instead of 2 mM CaCl_2_ where indicated. The LC_50_ was calculated as described in Materials and Methods. (C and N) Blots were probed with the indicated antibodies and horseradish peroxidase (HRP)–conjugated secondary antibodies. Graphs show the means ± SEM of at least (A, B, and D to G) three, (H to K) seven, (L and M) four, or (O to R) nine experiments. The blots show one representative experiment from (C) four or (N) six independent experiments. Data points represent individual experiments. Letters (a) to (c) denote statistically significant (*P* < 0.05) groups for each CDC using repeated-measures analysis of variance (ANOVA) between groups. *P < 0.05, **P < 0.01, and ***P < 0.001.

To determine whether the MEK/ERK pathway promoted repair during septic cardiomyopathy, we differentiated human induced pluripotent stem cells (iPSCs) into cardiomyocytes for an in vitro model of septic cardiomyopathy. We assessed cell death and contraction rate after SLO challenge during MEK inhibition with the well-established MEK inhibitor U0126. MEK inhibition in SLO-challenged iPSC-derived cardiomyocytes increased cell death (Fig. 1A). MEK inhibition also caused arrhythmia (increase in beats per minute) and uptake of the viability dye TO-PRO3, indicating cell dysfunction and death (Fig. 1B and movie S1). This suggests that MEK signaling promotes cell viability and regular contractile function during septic cardiomyopathy by mediating resistance to CDCs.

### Calcium-dependent plasma membrane repair relies on noncanonical MEK signaling

Since cells could resist CDCs either by reducing toxin binding (*27*) or by activating membrane repair (*1*, *3*), we tested both toxin binding and cell death in the genetically tractable HeLa cell lines, with and without MEK inhibition. U0126 blocked MEK activation (Fig. 1C and fig. S1, A and B), confirming inhibitor activity. CDC-specific MEK activation increased MEK and ERK phosphorylation above background, similar to the MEK activator platelet activating factor 16 (PAF16; Fig. 1C and fig. S1, A and B). To test the impact of U0126 on toxin binding, we used Cy5-conjugated toxins (*8*). Both active, wild-type (WT) SLO and nontoxic, SLO G398V/G399V “monomer-locked” (SLO ML) bound to HeLa cells in a dose-dependent manner, independently of U0126 treatment (Fig. 1, D to F). We conclude that increased cytotoxicity after MEK blockade is not due to changes in toxin binding to the membrane.

We next tested whether MEK signaling contributes to Ca^2+^-dependent membrane repair responses. We blocked MEK activity with U0126 with or without chelating extracellular Ca^2+^. We challenged HeLa cells with three different CDCs—SLO, PFO, or ILY—for 30 min and measured cytotoxicity by flow cytometry (Fig. 1, G to K). From these data, we calculated the toxin dose needed to lyse 50% of the cells [50% lethal concentration (LC_50_)] for each experimental condition to quantitate changes in cell susceptibility (*15*). To control for any impurities in the toxin preparation, we challenged cells with an equivalent mass of SLO ML (*8*, *28*). SLO ML did not cause cytotoxicity at any doses observed regardless of MEK inhibition (Fig. 1G). These data indicate that U0126 itself was not cytotoxic and lysis required toxin pore formation. Chelating Ca^2+^ with EGTA reduced all toxins’ LC_50_ by four- to fivefold (68 to 77% reduction; Fig. 1K), indicating that repair responses were blocked. Similarly, MEK blockade decreased the LC_50_ for PFO and SLO by ~3-fold (~60% reduction) and ILY by ~2-fold (45% reduction) (Fig. 1K). To determine whether MEK and Ca^2+^ chelation act in the same pathway, we combined U0126 treatment with EGTA. We found no additional decrease in LC_50_ for any CDC (Fig. 1K), indicating that MEK and Ca^2+^ signal in the same repair pathway. Since they act in the same pathway, MEK signaling contributed 80% of the Ca^2+^-dependent resistance to SLO and PFO, and 60% of the Ca^2+^-dependent resistance to ILY. We conclude that U0126 blocks most of the Ca^2+^-dependent repair responses to CDCs.

Since MEK inhibition reduces repair, we next determined whether MEK gain of function increased cellular resistance. We tested this hypothesis by treating SLO-challenged cells with the MEK activator PAF16. Induction of MEK using PAF16 increased cellular resistance ~1.5-fold (166% increase) (Fig. 1, L and M). Blockade of MEK activation using U0126 abrogated this increase, indicating that PAF16 increases cellular resistance via MEK activation. These data indicate that increasing MEK activity improves cellular resistance to SLO.

**Fig. 2.**
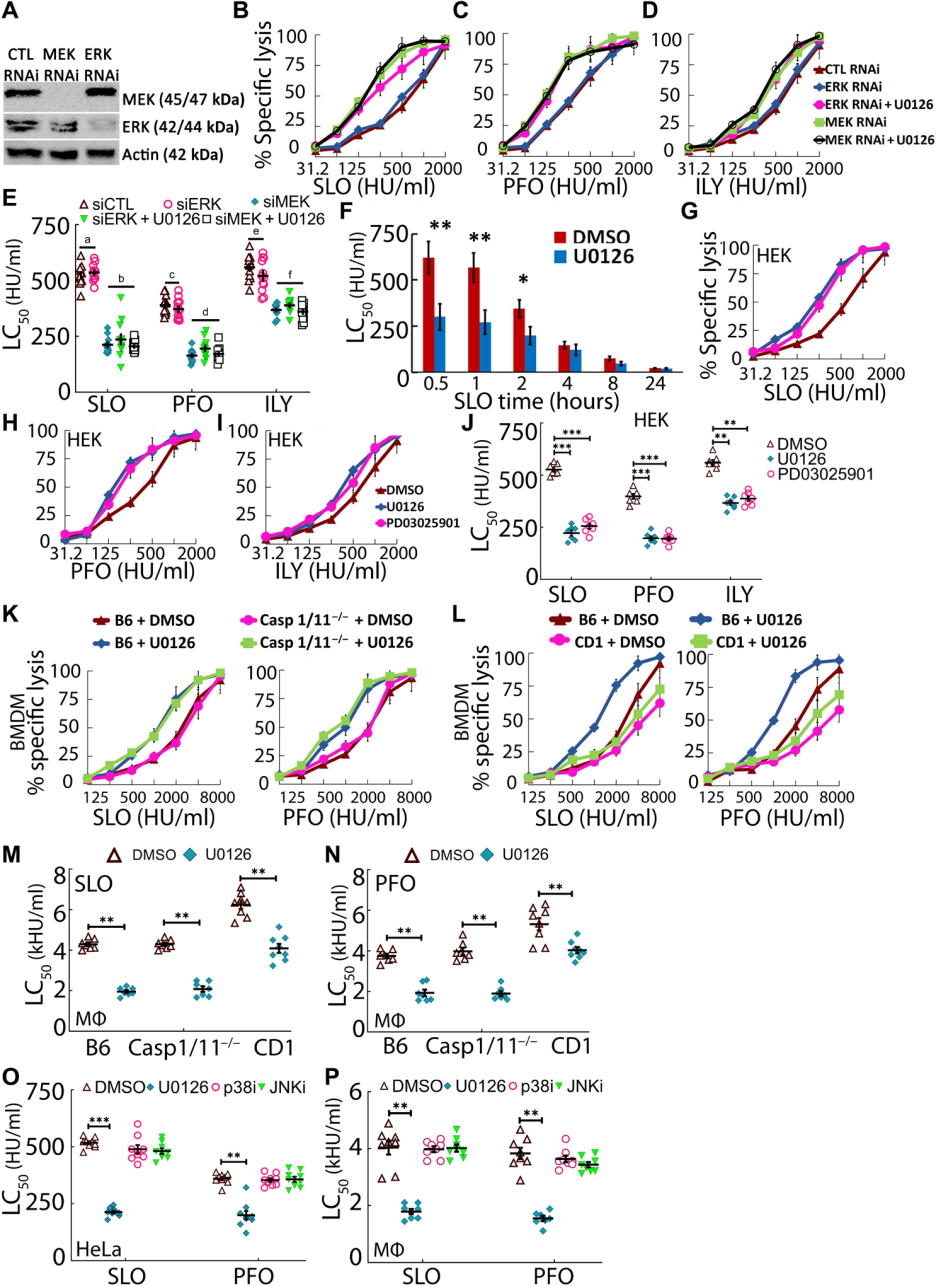
MEK reduces toxin-induced lysis independently of ERK. (**A** to **E**) HeLa cells were treated with control, MEK1/2, or ERK1/2 small interfering RNA (siRNA) for 72 hours and were (A) analyzed by Western blot. (B to E) RNAi-treated HeLa cells were pretreated with 20 μM MEK inhibitor U0126 or vehicle DMSO for 30 min and challenged with the indicated WT toxins at 31 to 2000 HU/ml for 30 min at 37°C. (**F**) HeLa cells were pretreated with DMSO or 20 μM inhibitor for 30 min, challenged with SLO at 31 to 2000 HU/ml, and assayed for viability by MTT assay at the indicated times. (**G** to **J**) HEK cells or (**K** to **N** and **P**) B6, Casp1/11^−/−^, or CD-1 BMDMs were pretreated with DMSO or 20 μM inhibitor for 30 min, challenged with the indicated WT toxins at (G to J) 31 to 2000 HU/ml or (K to N and P) 125 to 8000 HU/ml for 30 min at 37°C. JNK inhibitor (JNKi) is SP600125. p38 inhibitor (p38i) is SB203580. The LC_50_ was calculated as described in Materials and Methods. Blots were probed with the indicated antibodies and HRP-conjugated secondary antibodies. MΦ, macrophage. Graphs show the means ± SEM of at least (B to E) nine, (F) two, (G to N) seven, or (**O** and P) nine experiments. The blots show one representative experiment from at least six independent experiments. Data points represent individual experiments. Letters (a) to (f) denote statistically significant (*P* < 0.05) groups for each CDC using repeated-measures ANOVA between groups. *P < 0.05, **P < 0.01, and ***P < 0.001.

Since U0126 is an inhibitor, one hypothesis is that it acts via off-target effects. To test this hypothesis, we combined MEK inhibitors with RNA interference (RNAi). When HeLa cells were pretreated with other MEK inhibitors, PD3025901 or trametinib, and challenged with CDCs, repair responses were impaired similar to U0126 (Fig. 1, N to R). To further control for potential off-target effects of MEK inhibitors (*29*), we knocked down MEK1/2 or ERK1/2 by RNAi. RNAi gave 90% knockdown of MEK1/2 and ERK1/2 (Fig. 2A). When we challenged RNAi-treated cells with CDCs, MEK1/2 RNAi decreased LC_50_ similar to inhibitor treatment (Fig. 2, B to E). Unexpectedly, ERK1/2 RNAi did not decrease the LC_50_ (Fig. 2, B to E). To confirm the specificity of U0126 in our assays, we combined RNAi with U0126 treatment. When used in combination with U0126, MEK1/2 RNAi did not further decrease toxin LC_50_ (Fig. 2, B to E). These results indicate that U0126 is a specific inhibitor of MEK1/2 in our system. The combination of U0126 with ERK RNAi reduced the LC_50_ to that observed with MEK RNAi (Fig. 2, B to E), indicating ERK is not required for repair. We confirmed these results with the ERK-specific inhibitor magnolin (fig. S1, C to E). We also determined the protective window for MEK by measuring survival over time. MEK inhibition had the largest impact in the first hour of damage (Fig. 2F). We tested whether myoblast determination protein 1 (MyoD), which associates with MEK1 in the nucleus of differentiating myoblasts (*30*) was phosphorylated after CDC intoxication. Neither MEK inhibition with U0126 nor toxin challenge changed levels of phosphorylated MyoD. Overall, RNAi validated the inhibitors in our system, so we conclude that MEK signals in an ERK-independent manner to promote membrane repair in HeLa cells.

We next extended our analysis of MEK-dependent repair to other cell types. Since U0126 was validated by RNAi, different cell types show variable transfection efficiencies, and U0126 could be used as a therapy; we used U0126 in subsequent assays. In human embryonic kidney (HEK) cells, MEK inhibition with U0126 or PD3025901 reduced CDC LC_50_ similar to HeLa cells (Fig. 2, G to J). Primary macrophages are 10 to 20 times more resistant than HeLa cells (*8*, *31*), so we tested whether this increased resistance was due to MEK. We compared bone marrow–derived macrophages (BMDMs) from WT C57BL/6 (B6) and Caspase 1/11 (Casp1/11)^−/−^ mice to rule out any role for toxin-induced pyroptosis (*31*). MEK inhibition with U0126 (Fig. 2, K to N) increased BMDM sensitivity by twofold (~50% reduction) regardless of Casp1/11, but BMDMs remained more resistant to CDCs than HeLa and HEK cells. These data indicate that BMDMs use MEK-dependent repair but that their additional resistance is not due to MEK. However, B6 mice contain a cryptic splice donor site that truncates the repair protein A6 (*32*). Since A6 is both a repair protein and a marker for membrane repair (*14*, *15*, *32*), we tested the impact of full-length A6 on MEK-dependent repair using the outbred CD-1 strain. CD-1 BMDMs were ~1.4-fold more resistant (140% increase) than their B6 counterparts (Fig. 2, L to N), potentially due to full-length A6. However, upon MEK inhibition, the SLO and PFO LC_50_ in CD-1 BMDMs was reduced by ~1.5-fold (~30% reduction), compared to the ~2-fold reduction (50% reduction) in B6 BMDMs (Fig. 2, L to N). We conclude that MEK-dependent repair constitutes a majority of calcium-dependent repair across several cell types.

Since p38 and JNK are implicated in long-term viability after toxin challenge, we tested whether they contribute to membrane repair. The p38 and JNK inhibitors blocked phosphorylation of p38 (fig. S1F) and JNK (fig. S1G). However, neither p38 nor JNK inhibition altered repair responses to CDCs in HeLa cells or BMDMs (Fig. 2, O and P, and fig. S1, H to K). We conclude that proximal membrane repair responses are specific to MEK activation.

### MLKs activate MEK-dependent repair

**Fig. 3.**
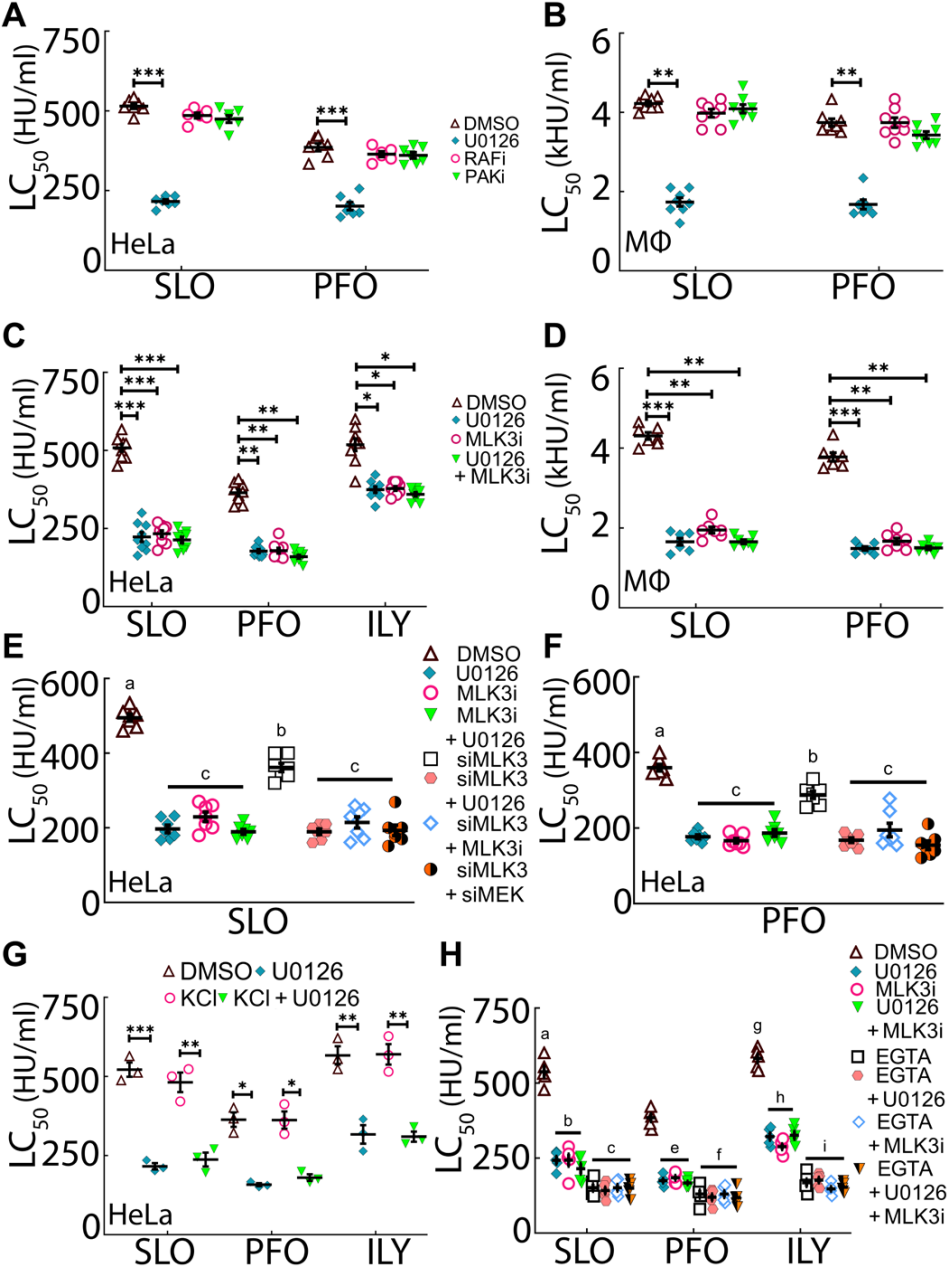
MLKs activate the MEK-dependent repair pathway. HeLa cells (**A**, **C**, and **E** to **H**) or B6 BMDMs (**B** and **D**) were pretreated with DMSO or 20 μM inhibitor(s) for 30 min and challenged with the indicated WT toxins at (A, C, and E to H) 31 to 2000 HU/ml or (B and D) 125 to 8000 HU/ml for 30 min at 37°C, and PI uptake was analyzed by flow cytometry. (E, **F**, and H) HeLa cells were treated with control, MEK1/2, and/or MLK3 siRNA for 72 hours before the assay described above. In (**G**), 150 mM KCl was included during the assay. In (H), 2 mM EGTA was used instead of 2 mM CaCl_2_ where indicated. PAK inhibitor (PAKi) is FRAX597, RAF inhibitor (RAFi) is LY3009120, and MLK3 inhibitor (MLK3i) is URMC-099. The LC_50_ was calculated as described in Materials and Methods. Graphs show the means ± SEM of at least (A and B) seven, (C) nine, (D to F) seven, (G) three, or (H) five experiments. Data points represent individual experiments. Legends between panels describe both panels. Letters (a) to (i) denote statistically significant (*P* < 0.05) groups for each CDC using repeated-measures ANOVA between groups. For example, in (E) and (F), *P* < 0.05 for MLK3 siRNA compared to group c and *P* < 0.01 compared to DMSO, while DMSO was *P* < 0.01 for MLK3 siRNA and *P* < 0.001 compared to all other groups. *P < 0.05, **P < 0.01, and ***P < 0.001.

We next determined the kinase that activates MEK during membrane repair. Since several kinases that phosphorylate MEK are implicated in toxin resistance, we screened kinase pathways with broad-spectrum inhibitors. To test the role of RAF and PAK kinases in MEK-dependent repair, we used pan-Raf (LY3009120) and PAK (FRAX597) inhibitors to broadly block RAF or PAK before challenge with SLO and PFO (Fig. 3, A and B, and fig. S2, A to D). Neither RAF nor PAK blockade sensitized HeLa cells (Fig. 3A and fig. S2, A and B) or BMDMs (Fig. 3B and fig. S2, C and D) to CDCs compared to dimethyl sulfoxide (DMSO)–treated controls. We conclude that RAF and PAK do not mediate MEK-dependent membrane repair responses.

We next tested MLK3 because MLK3 phosphorylation of MEK can decouple it from ERK (*26*). We used the therapeutic MLK inhibitor URMC-099 (*33*) to determine the impact of MLK on MEK-dependent repair. URMC-099 blocked MEK and ERK phosphorylation (fig. S2E). When we pretreated HeLa cells, HEK cells, WT BMDMs, or Casp1/11^−/−^ BMDMs with URMC-099 or U0126 and challenged them with CDCs, we found that URMC-099 reduced LC_50_ of CDCs compared to DMSO controls, similar to U0126 (Fig. 3, C and D, and fig. S2, F to Q). Furthermore, when URMC-099 and U0126 were used in combination (Fig. 3, C and D, and fig. S2, F to Q), no additional increase in cytotoxicity was observed. These data suggest that MEK-dependent repair is triggered by MLKs.

Since URMC-099 is a broad-spectrum inhibitor with multiple targets (*33*), including MLK1, MLK2, MLK3, dual leucine zipper-bearing kinase, and leucine-rich repeat kinase, we knocked down MLK3 using RNAi to determine whether MLK3 drives repair. RNAi gave ~80% knockdown of MEK, ERK, and MLK3 (fig. S3A). Cells treated with MLK3 RNAi had a ~1.5-fold decrease (~30% reduction) in CDC LC_50_ compared to control RNAi-treated cells, which was less than that in MEK RNAi-, U0126-, or URMC-099–treated cells (Fig. 3, E and F, and fig. S3B). When combined with MEK RNAi, U0126, or URMC-099, no additional effects were observed from MLK3 RNAi (Fig. 3, E and F, and fig. S3B), confirming that MLK3 acts in the MEK-dependent repair pathway. We interpret these data to indicate that MLKs, including MLK3, redundantly activate MEK to promote MEK-dependent repair.

### MLK3 is activated independently of PKC and ceramide

There are several possible upstream signaling molecules that could activate MLK3 during CDC attack. Potassium efflux may trigger MEK during LLO challenge (*9*, *34*) via an unknown mechanism. Alternatively, calcium influx activates PKC, which activates MLK3 in stress responses (*35*). Last, sphingomyelinase activity during CDC attack may generate ceramide (*23*, *24*), which can activate MLK3 in flies and humans (*25*, *26*). To determine which of these mechanisms drive MEK-dependent repair, we sequentially blocked each pathway. To prevent K^+^ efflux, HeLa cells with or without U0126 were challenged with CDCs in the presence of 150 mM KCl. Extracellular KCl did not lower CDC LC_50_ (Fig. 3G) or alter the CDC dose-response curve (fig. S3C), despite higher levels of phospho-ERK and MEK (fig. S3D). We interpret these data to conclude that K^+^ efflux does not trigger MEK-dependent repair.

We next tested the role of calcium in MLK3-dependent repair. We combined Ca^2+^-chelation with URMC-099 or U0126 and determined the CDC LC_50_ in HeLa cells. We found that URMC-099 phenocopied U0126 and did not provide additive effects to EGTA treatment (Fig. 3H and fig. S3E). Treatment of SLO-challenged cells with EGTA reduced MLK3 phosphorylation to baseline (fig. S3F). These data confirm that MLK3 is phosphorylated after Ca^2+^ influx and acts in the same pathway as Ca^2+^ influx to promote MEK-dependent repair. Since Ca^2+^ influx activates PKC and PKC can activate MLK3, we tested whether PKC was involved in MEK-dependent repair using the pan-PKC inhibitor Go6983. We found a reduction in the PFO LC_50_, but not SLO LC_50_, upon PKC inhibition (fig. S3, G and H). When used in combination with U0126 or URMC-99, Go6983 did not have any additive effects on cytotoxicity (fig. S3, G and H). Since PFO requires more time to become cytotoxic than SLO owing to their differences in binding affinities (*15*, *36*), it is possible that this difference underlies the different CDC responses to PKC inhibition. We interpret these results to indicate that PKC is not substantially involved in MEK-dependent repair.

**Fig. 4.**
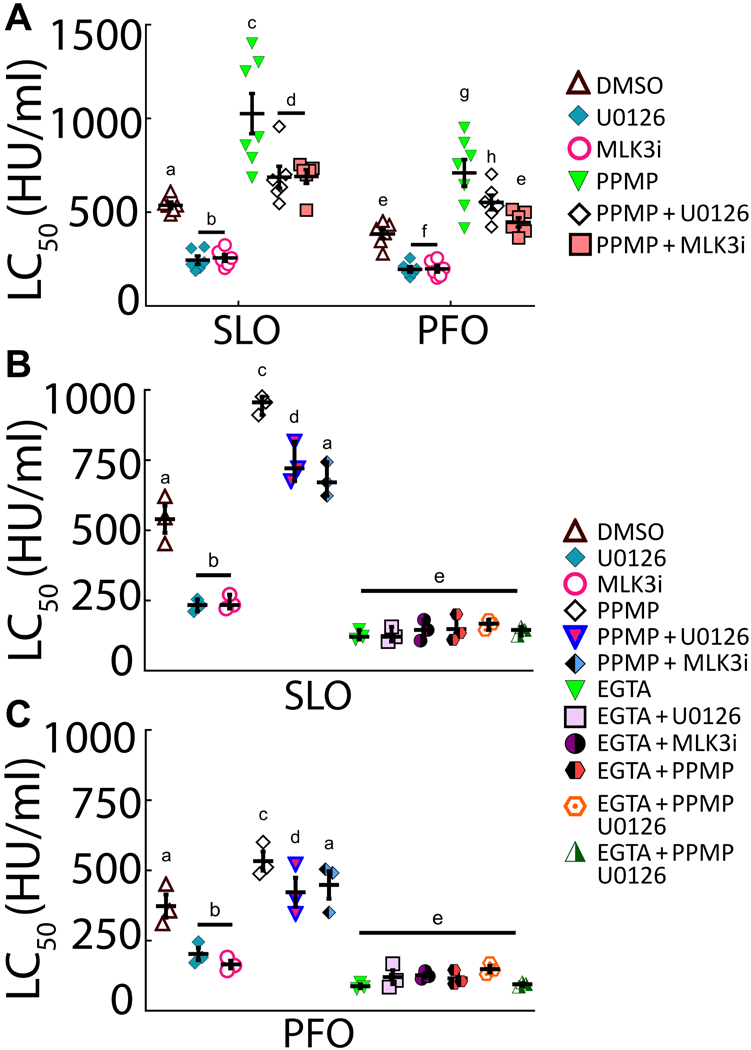
Ceramide may activate a parallel repair pathway. HeLa cells were pretreated with 40 μM PPMP for 48 hours and then DMSO or 20 μM inhibitor for 30 min and challenged with (**A** and **B**) SLO or (A and **C**) PFO at 31 to 2000 HU/ml for 30 min at 37°C, and PI uptake was analyzed by flow cytometry. In (B) and (C), 2 mM EGTA was used instead of 2 mM CaCl_2_ where indicated. The LC_50_ was calculated as described in Materials and Methods. Graphs show the means ± SEM of at least (A) seven or (B and C) three experiments. Data points represent individual experiments. Letters (a) to (h) denote statistically significant (*P* < 0.05) groups for each CDC using repeated-measures ANOVA between groups.

Since sphingomyelinase is implicated in membrane repair (*23*, *24*) and ceramide can activate MLK3 (*25*, *26*), we tested the hypothesis that ceramide triggers MLK3 and MEK to promote membrane repair. To distinguish between sphingomyelin depletion and ceramide elevation, we used d-threo-1-phenyl-2-hexadecanoylamino-3-morpholino-1-propanol ·HCl (PPMP) for 72 hours to promote ceramide accumulation in cells (*37*). We used SLO ML Cy5, ostreolysin A (OlyA) E69A–mCherry, and WT OlyA-mCherry to determine whether PPMP altered CDC-accessible cholesterol, total sphingomyelin levels, or cholesterol-sphingomyelin complexes, respectively (*38*, *39*). PPMP did not alter these lipids (fig. S4A). We then determined whether ceramide signals through the MLK3/MEK axis during CDC challenge. PPMP made the cells ~2-fold more resistant (200% increase) to CDCs compared to control _(_Fig. 4A and fig. S4B), corroborating the previously established protective role of ceramide (*14*, *40*). While U0126 or URMC-99 reduced the CDC LC_50_ of PPMP-treated cells, it did not fully overcome the enhanced protection provided by PPMP (Fig. 4A and fig. S4, C and D). Instead, MEK or MLK3 inhibition only overcame ~60% of PPMP-mediated resistance in cells (Fig. 4A). We next determined whether PPMP-mediated toxin resistance was calcium dependent and whether it acted in the same pathway as MEK and MLK3 by combining Ca^2+^ chelation with PPMP and MEK/MLK inhibition during CDC challenge. While PPMP provided ~2-fold protection (~200% increase) during CDC challenge, Ca^2+^ chelation rendered PPMP-treated cells equally sensitive to non-PPMP–treated cells (Fig. 4, B and C, and fig. S4, E and F). MEK or MLK3 inhibition only partly sensitized PPMP-treated cells but showed no additive effects beyond Ca^2+^ chelation (Fig. 4, B and C). We conclude that MLK3/MEK-dependent protection and ceramide-mediated protection may be parallel, Ca^2+^-dependent repair pathways.

### MEK delays Ca^2+^ influx

**Fig. 5.**
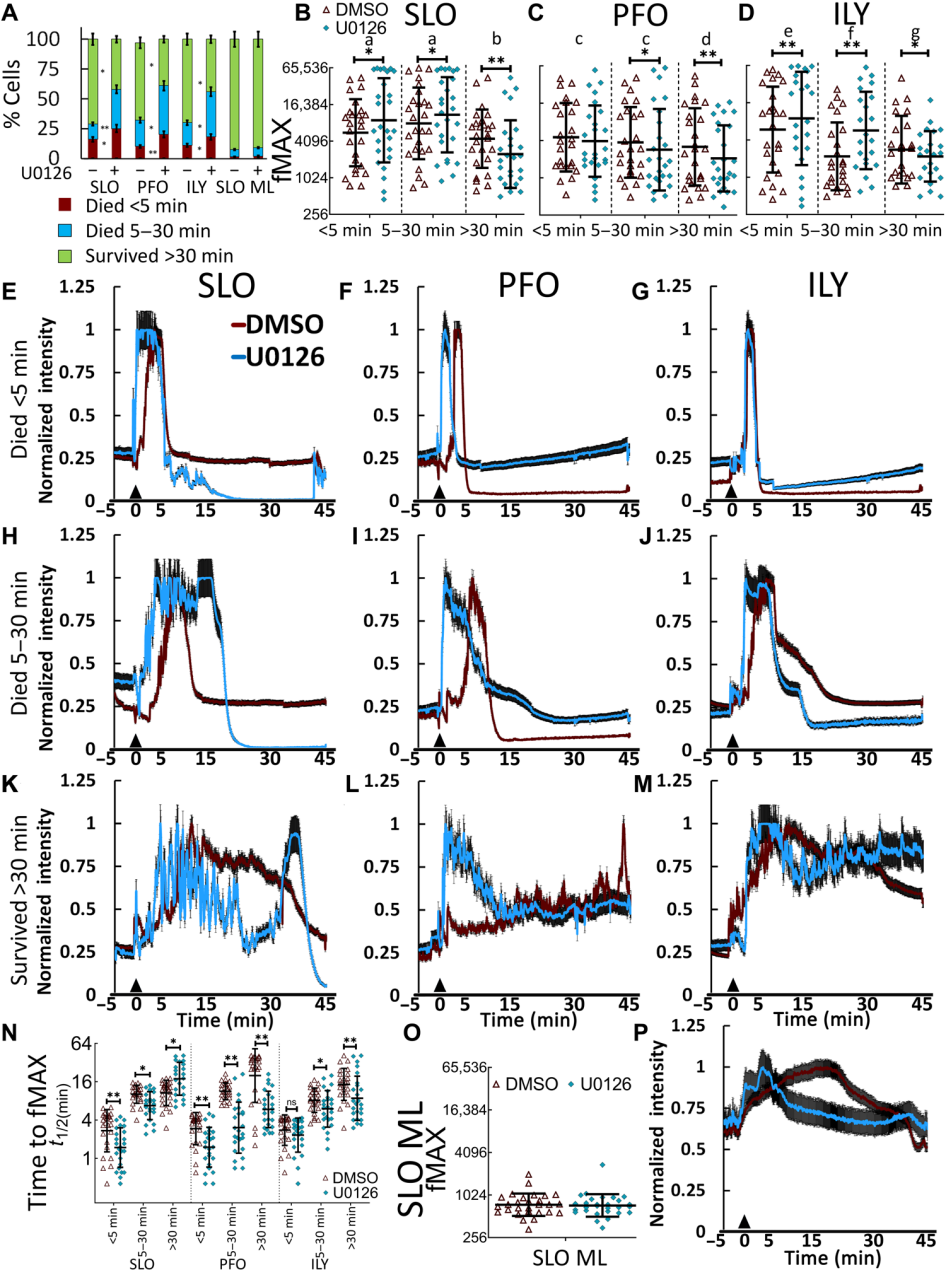
MEK prevents rapid Ca^2+^ influx. Cells were incubated with Fluo-4 and DMSO or 20 μM U0126 for 30 min. The medium was replaced with TO-PRO3 (2 μg/ml), 25 mM Hepes (pH 7.4), and 2 mM CaCl_2_ (imaging buffer), and sublytic toxin was added 5 min after confocal imaging started. Cells were imaged for ~45 min at 37°C. Calcium flux and cell death were recorded. Cells were categorized into three subsets by time of cell death: died <5 min, died between 5 and 30 min, and survived >30 min. (**A**) The percentage of each subset is shown. (**B** to **D** and **O**) Maximal Fluo-4 fluorescent intensities (fMAX) are shown for individual cells. (**E** to **M** and **P**) Fluo-4 fluorescence intensity was normalized to fMAX for individual cells and averaged. Calcium traces for (E to G) cells that died <5 min, (H to J) cells that died between 5 and 30 min, or (K to M and P) cells that survived >30 min after challenge with (E, H, and K) SLO (250 HU/ml), (F, I, and L) PFO (125 HU/ml), (G, J, and M) ILY (250 HU/ml), or (P) SLO ML (20 μg/ml) challenge. (**N**) The time to reach fMAX is shown. Data points represent individual cells from at least three independent experiments. Graphs show the geometric means ± SD of at least three independent experiments. Letters (a) to (g) denote statistically significant (*P* < 0.05) groups using repeated-measures ANOVA between each toxin in (B) to (D) to compare differences in fMAX between 5 min, 5 to 30 min, and survivors. *P < 0.05 and **P < 0.01. ns, not significant.

Since calcium overload is one mechanism by which toxins kill cells (*41*), we measured calcium influx and viability in CDC-challenged cells with and without MEK-dependent repair. To analyze Ca^2+^ flux, we categorized cells based on cell death. One subset died within 5 min of CDC challenge. A second subset died between 5 and 30 min of CDC challenge. All remaining cells fit in the third subset, which survived the entire 45-min experiment. MEK inhibition increased both subsets that died (Fig. 5A and movies S2 and S3). We compared the maximum calcium influx for each subset with or without U0126, using all three CDCs. Cells that died from SLO had the highest Ca^2+^ levels, while PFO had the lowest (Fig. 5, B and C). Cells that died from SLO or ILY had higher maximal Ca^2+^ levels than surviving cells (Fig. 5, B and D). While U0126 increased maximal Ca^2+^ influx in cells that died from SLO and ILY, surviving cells had lower maximal Ca^2+^ (Fig. 5, B to D). These data suggest that parallel Ca^2+^-dependent pathways dictate survival in the absence of MEK signaling.

We next compared Ca^2+^ flux with and without MEK inhibition in each group of cells. Cells that died within 5 min of toxin challenge rapidly peaked Ca^2+^ (Fig. 5, E to G), which was significantly faster in MEK-inhibited cells challenged with SLO or PFO, but not ILY (Fig. 5, E to G). U0126-treated cells that died after 5 min reached peak Ca^2+^ intensity earlier than the DMSO-treated cells regardless of CDC used (Fig. 5, H to J). In contrast to cells that died, surviving cells fluctuated Ca^2+^ levels for the entire imaging period (Fig. 5, K to M, and movies S2 and S3). MEK inhibition drove a more rapid influx than DMSO in surviving cells challenged with PFO and ILY, but a slower response to SLO (Fig. 5, K to N). The nontoxic SLO ML did not promote Ca^2+^ flux at any time points (Fig. 5, O and P, and movie S2). These data suggest that MEK slows Ca^2+^ influx by promoting early repair responses.

### MEK promotes repair by increasing microvesicle shedding

**Fig. 6.**
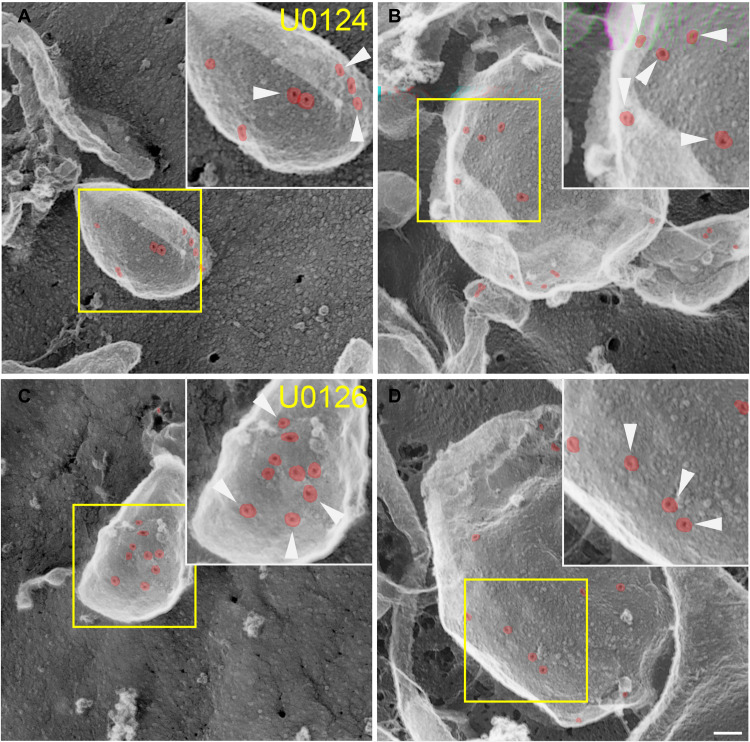
MEK is not required for SLO sequestration on blebs. HeLa cells were pretreated with (**A** and **B**) 20 μM inactive analog U0124 or (**C** and **D**) 20 μM U0126 for 30 min and challenged with a sublytic dose of SLO for 5 min at 37°C, washed, fixed in 2% glutaraldehyde, and analyzed by rapid-freeze, deep-etch EM. Arrowheads mark SLO pores, which are pseudo-colored red to aid visualization. Micrographs show representative portions of cells from one of three independent experiments. Scale bar, 100 nm.

Since microvesicle shedding is the primary repair mechanism against bacterial CDCs (*5*, *8*, *42*), we determined which step of shedding was controlled by MEK activation. During microvesicle shedding, CDCs are first clustered on blebs and then shed (*5*). We used rapid-freeze, “deep-etch” electron microscopy (EM) to determine whether SLO clustering on blebs was reduced upon MEK inhibition (Fig. 6). We observed no difference in SLO sequestration on blebs, regardless of MEK inhibition (Fig. 6). We counted at least 100 pores from U0124- and U0126-treated cells and found that 118 of 121 and 114 of 118, respectively, were localized to blebs. We conclude that MEK does not reduce toxin sequestration on blebs.

**Fig. 7.**
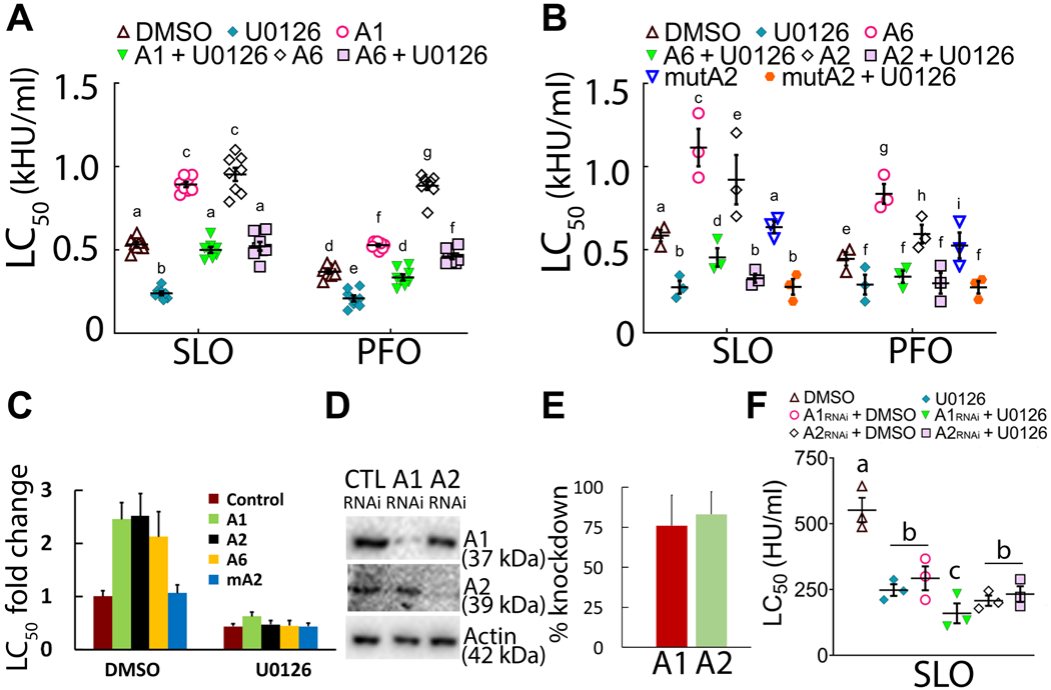
Annexins enhance cellular resistance to toxins. HeLa cells were transfected with control, fluorescent annexins, or siRNAs to annexins, treated with DMSO or 20 μM U0126 for 30 min, and challenged with SLO or PFO at 31 to 2000 HU/ml for 30 min at 37°C, and PI uptake was analyzed by flow cytometry, or knockdown efficiency was evaluated by Western blot. (**A** to **C**) HeLa cells were untransfected or transfected with (A and C) A1-YFP, (A to C) A6-YFP, (B and C) A2-GFP, or (B and C) mutant A2-GFP (mutA2) for 48 hours. (**D** to **F**) HeLa cells were transfected with siRNA against A1 or A2 for 72 hours and analyzed by (D and E) Western blot or (F) flow cytometry. Blots were probed with the indicated antibodies and HRP-conjugated secondary antibodies and quantitated to assess annexin knockdown. The LC_50_ was calculated as described in Materials and Methods. Graphs show the means ± SEM of at least (A) eight, (B, F) three, or four (E) experiments. (C) Fold changes in LC_50_ for (A) and (B) were calculated. The blot shows one representative experiment from four independent experiments. Data points represent individual experiments. Letters (a) to (h) denote statistically significant (*P* < 0.05) groups for each CDC using repeated-measures ANOVA between groups.

Since MEK does not alter sequestration, it might control microvesicle shedding. To test this hypothesis, we visualized shedding with fluorescent annexins. Since annexins are also Ca^2+^-dependent repair proteins, we first tested the impact of expressing fluorescent annexins in cells on repair. The transfection efficiency of HeLa cells with fluorescently tagged annexins was 65 to 80% (fig. S5A). We found that fluorescently tagged A1, A6, and A2 increased cellular resistance to CDCs by ~2-fold compared to control cells (166 to 250% increase) (Fig. 7, A and B, and fig. S5, B to E). However, a mutant A2 (mutA2) that could not bind Ca^2+^ did not increase cellular resistance, ruling out potential overexpression artifacts as the source of cellular resistance (Fig. 7B and fig. S5, D and E). Annexin expression did not alter CDC-accessible cholesterol, total sphingomyelin, or cholesterol-sphingomyelin complex levels (fig. S5, F and G). MEK inhibition reduced the CDC LC_50_ of annexin-expressing cells, but it did not fully overcome the enhanced protection provided by A1 or A6 (Fig. 7, A and B). To confirm the change in protection, we compared the fold change in LC_50_ (Fig. 7C). We found cells that expressed fluorescent annexins were roughly ~2-fold more resistant (200% more resistant) to toxins. In contrast to A1 and A6, A2 failed to enhance SLO resistance in cells treated with U0126 (Fig. 7B and fig. S5, D and E). This suggests that A2 may be downstream of MEK, while A1 and A6 appeared to act in a parallel pathway. All three annexins acted in Ca^2+^-dependent repair (Fig. 7, A and B, and fig. S5, H and I). Overall, these data indicate that A1 and A6 can serve as markers of repair and microvesicle shedding in our system.

We next confirmed the role of annexins in membrane repair by knocking them out. We depleted A1 or A2 by small interfering RNA (siRNA; Fig. 7, D and E). We then challenged the cells with SLO in the presence or absence of U0126. We found that knockdown of A1 or A2 alone increased the cell sensitivity to SLO (Fig. 7F). When combined with U0126, A1 knockdown showed an additive effect on increasing cell death (Fig. 7F). In contrast, no additional additive effects were observed with A2 and U0126. These data further suggest that A2 acts in the same pathway as MEK, whereas A1 may not.

**Fig. 8.**
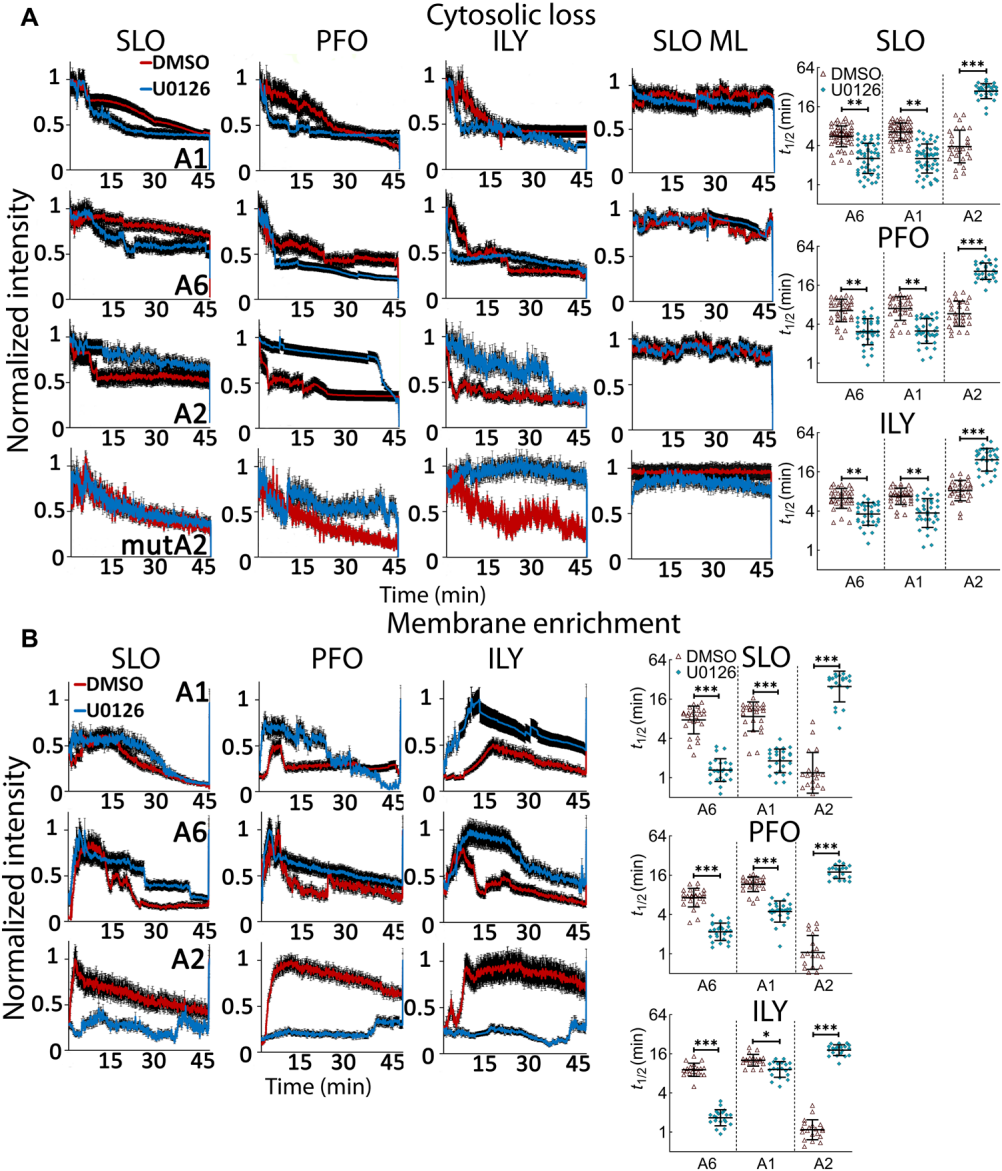
MEK controls the rate of A2 membrane recruitment. A1-YFP–, A6-YFP–, A2-GFP–, or mutA2-GFP–transfected HeLa cells were challenged with SLO, PFO, ILY, or SLO ML in imaging buffer after 30-min pretreatment with DMSO or 20 μM U0126. The cells were immediately imaged by confocal microscopy for ~45 min at 37°C and then treated with 1% Triton X-100 to end the experiment. (**A**) The average annexin cytoplasmic depletion or (**B**) membrane enrichment over time was normalized to maximal intensity and *t*_1/2_ calculated. Traces display the means ± SEM of at least three independent experiments. Scatterplots show the geometric means ± SD. Data points represent individual cells. *P < 0.05, **P < 0.01, and ***P < 0.001.

**Fig. 9.**
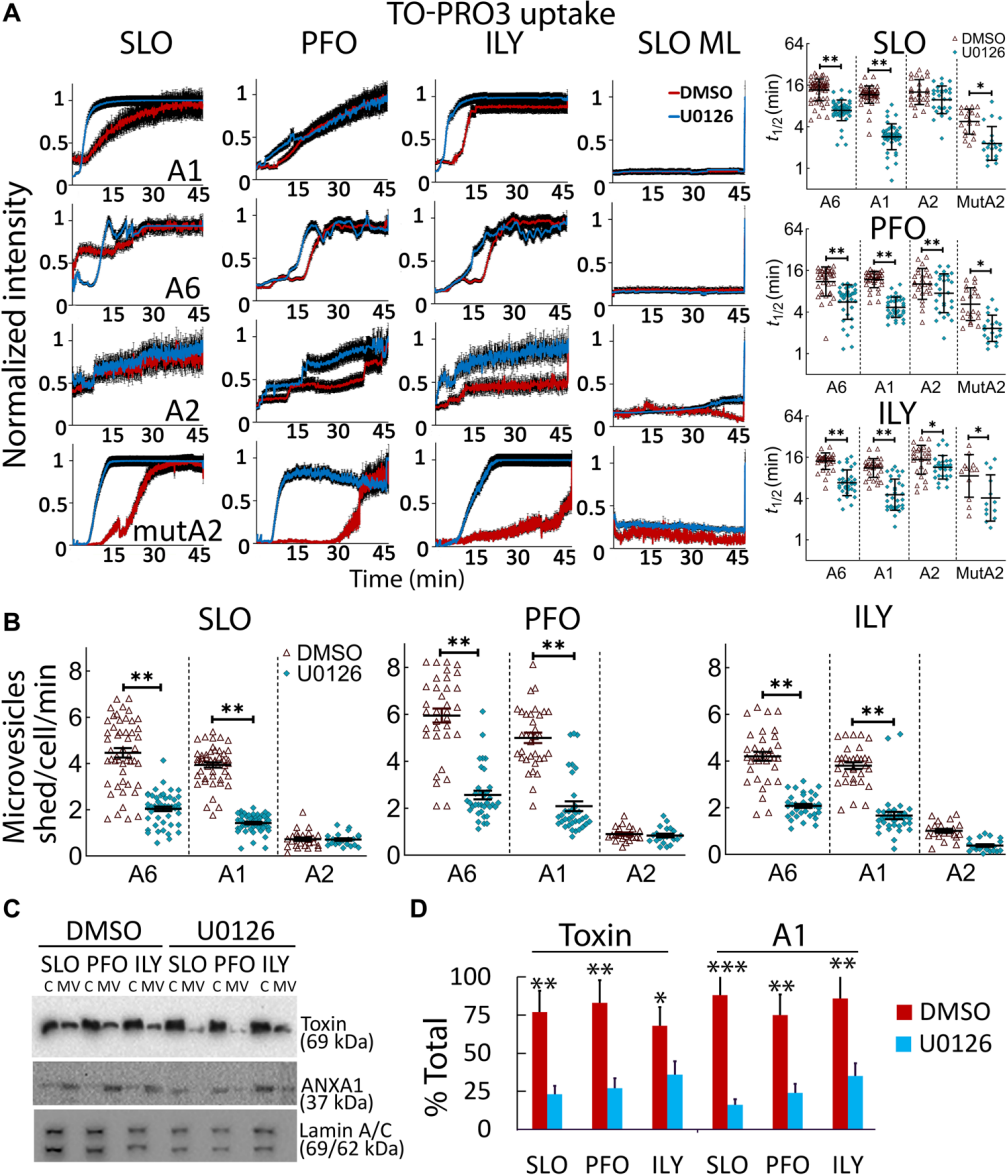
MEK promotes survival by recruiting A2 to the site of damage and enhancing microvesicle shedding. A6-YFP–, A1-YFP–, A2-GFP–, or mutA2 GFP–transfected HeLa cells from Fig. 8 were analyzed for (**A**) TO-PRO3 uptake, or (**B**) shedding of annexin + microvesicles. (**C** and **D**) HeLa cells were pretreated for 30 min with DMSO or 20 μM U0126 and challenged with sublytic SLO, PFO, or ILY for 15 min at 37°C. Cells were pelleted at 2000*g* for 5 min to yield cell pellet (C). Cell supernatants were spun at 100,000*g* for 40 min at 4°C and microvesicle (MV) pellet collected. (C) Fractions were analyzed by Western blot using 6D11 anti-SLO, CPTC-A1-3 anti-A1, or MANLAC-4A7 anti–Lamin A/C. (D) The fraction of toxin or A1 present in the MV pellet was quantitated. Traces display the means ± SEM of at least three independent experiments. Scatterplots show geometric means ± SD. Data points represent individual cells from at least three independent experiments. The blots show one representative experiment from four independent experiments, which were quantitated in (D). *P < 0.05, **P < 0.01, and ***P < 0.001.

We measured repair by tracking annexin movement from the cytosol to the membrane during CDC challenge by live-cell imaging. Annexin membrane recruitment can be measured by the quantitative depletion of annexins from the cytosol (Fig. 8A) or local recruitment to the membrane (Fig. 8B), while cell permeabilization and cell death can be measured by degree of TO-PRO3 uptake (Fig. 9A and movies S4 to S10). Our results were similar to our previous results (*15*). Our results differ from a previous report that showed a much faster recruitment of annexins and A6 cycling (*14*). This is likely due to the elevated Ca^2+^ we observed in surviving cells (Fig. 5, K to M). SLO ML did not cause any annexin translocation (Fig. 8 and movies S4, S6, and S7). In cells challenged with WT SLO, PFO, or ILY, MEK inhibition hastened the cytoplasmic depletion of both A6 and A1 (Fig. 8A and movies S4 to S6). The half-time (*t*_1/2_) for A6 and A1 depletion was ~2.5-fold lower (~60% reduction) after MEK inhibition for SLO, PFO, and ILY (Fig. 8A). This quantitative depletion was accompanied by faster accumulation of A6 and A1 at the membrane (Fig. 8B and movies S4 to S6). The *t*_1/2_ of A6 and A1 membrane recruitment was ~8 to 10 min in DMSO-treated control compared to 1 to 3 min for MEK inhibited cells (Fig. 8B). Since annexin depletion does not measure viability or permeability, we analyzed TO-PRO3 uptake. During toxin challenge, cells may take up small amounts of dye without dying (*15*). MEK inhibition accelerated dye uptake, with a ~2-fold reduction (~50% reduction) in *t*_1/2_ across CDCs and annexins (Fig. 9A). We interpret these data to suggest that repair defects in MEK-inhibited cells leads to a rapid rise in intracellular Ca^2+^ concentration that drives a rapid, parallel A1 and A6 repair pathway in an attempt to limit the damage.

To determine the contribution of MEK to microvesicle shedding in annexin-transfected cells, we quantitated annexin shedding. We found that MEK inhibition generally reduced the shedding of A6–yellow fluorescent protein (YFP) and A1-YFP by ~2- to 3-fold across CDCs (~50 to 65% reduction) (Fig. 9B and movies S4 to S6). We interpret these data as showing that A1 and A6 are passengers on microvesicles and that they do not contribute to resistance provided by microvesicle shedding. This indicates that A1 and A6 are insufficient to promote shedding and that they act to promote repair by another mechanism (such as clogging). In contrast to previous results (*10*), we observed very little A2–green fluorescent protein (GFP) shedding in DMSO-treated toxin-challenged cells, which was not altered by MEK inhibition (Fig. 9B and movies S7 and S8). Overall, translocation was similar among A1, A2, and A6, regardless of CDC used or MEK inhibition (fig. S5J). To confirm microvesicle shedding, we measured microvesicle shedding by ultracentrifugation and Western blot, as we have previously done (*8*). Consistent with a reduction in microvesicle shedding, we observed a reduction in SLO and A1 in the microvesicle pellet upon MEK blockade (Fig. 9, C and D). The nuclear proteins Lamin A/C remained in the cell pellet after CDC challenge, confirming the sublytic dose and the absence of cellular debris in the microvesicle fraction (Fig. 9C). Overall, these data suggest that MEK mediates membrane repair in response to CDCs by driving microvesicle shedding.

### MEK recruits A2 to the membrane to promote repair

In contrast to A1 and A6, A2 recruitment to the membrane was delayed by MEK inhibition. Quantitative depletion of A2 was ~8 to 10 times slower upon MEK inhibition for all CDCs (Fig. 8A and movies S7 and S8). A2 membrane enrichment switched from being the first annexin on the surface in control cells (*t*_1/2_ ~ 1 min) to the slowest annexin in MEK-inhibited cells (*t*_1/2_ ~ 25 to 30 min; Fig. 8B and movies S7 and S8). To control for potential transfection artifacts, we measured mutA2 responses to CDCs by live-cell imaging. We observed no membrane enrichment or vesicle shedding and only a negligible difference in cytosolic depletion with mutA2 (Fig. 8 and movies S9 and S10). These data suggest that A2 could be one potential target for MEK and that the early recruitment of A2 is critical to limiting Ca^2+^ influx, initiating membrane repair, and promoting cell survival. We propose a model where A2 is recruited to the membrane by MEK. At the membrane, A2 promotes repair by driving microvesicle shedding. However, as an early component of repair, A2 itself is not shed in the vesicles whose formation it catalyzes. In contrast, A1 and A6 are recruited by MEK-independent mechanisms, where they promote repair by a complementary mechanism, such as forming a crystalline array that effectively clogs the pore. While the A1 and A6 crystalline arrays are not required for shedding, they are shed along with toxin. Thus, A1 and A6 are markers of shedding, but not responsible for shedding themselves, whereas A2 promotes shedding but is minimally present on shed vesicles.

## DISCUSSION

In this study, we demonstrate a previously unidentified signaling mechanism that mediates the majority of Ca^2+^-dependent repair responses to bacterial CDCs. We find that during membrane repair, calcium influx activates MLK3, which, in turn, uncouples MEK from ERK. ERK-independent MEK signaling promotes rapid A2 membrane recruitment and enhances microvesicle shedding. Notably, potassium efflux does not activate repair, while ceramide and A1 and A6 act in parallel, Ca^2+^-dependent pathways that do not compensate for a failure of protective MEK signaling. These findings expand the prosurvival role of MEK to include membrane repair by membrane shedding and suggest MEK may protect cells during NSTIs and septic cardiomyopathy.

Since there are several pathways by which Ca^2+^ influx could activate MEK and ERK MAPK, it was unclear which pathways, if any, triggered membrane repair. Many of these pathways signal via Ras, Raf, or PAK (*21*, *43*), none of which were necessary for Ca^2+^-dependent repair. Instead, we find that MLKs are necessary for repair. While MLK3 is the primary MLK to contribute to repair, we observe some redundancy with other URMC-099–inhibited kinases. We interpret these results to indicate redundancy between MLK3 and other MLKs. This is not unusual because MLKs have differential tissue expression. The activation of MLKs during repair remains an open question. While PKC and ceramide can both activate MLK3 (*25*, *26*, *35*), they did not activate MLK3 during repair. Both Rac and cdc42 can also activate MLK3 (*44*, *45*). This could represent one mechanism by which MLK3 is activated. Since Rac and cdc42 are recruited during repair (*46*), these small guanosine triphosphatases could provide a coordinated response to membrane damage. However, separating the impacts of Rac and cdc42 on the cytoskeleton during repair from their impact on MLK3 during repair is not trivial. Alternatively, steady-state phosphorylation of MLK3 and MEK could be needed to keep the repair system “primed” for repair. Future studies are needed to determine whether cdc42 directly activates MLK3 after toxin challenge or whether an as-yet-unknown pathway activates MLK3 downstream of Ca^2+^.

While MLK3 signaling is associated with the decoupling of MEK and ERK in other systems (*26*), ERK was previously thought to act in membrane repair. ERK signaling to mortalin was the interpretation of increased mortality due to MEK inhibition during membrane attack complex (MAC) killing of cells (*47*). We suggest that MAC may instead trigger the same ERK-independent pathway as CDCs. Since anthrax lethal toxin targets MEK for destruction (*48*), our findings further suggest one purpose in targeting MEK may be to cripple membrane repair to sensitize cells to the *Bacillus anthracis* CDC anthrolysin O. We suggest an unidentified MEK target may be activated only when MLKs activate MEK. Since upstream MEK kinases converge on Ser^217^ and Ser^221^, our work raises the question of how MLKs uncouple MEK from ERK, while other MEK kinases do not. MLKs could target other parts of MEK for phosphorylation, or they could target an as-yet unidentified accessory protein not targeted by other kinases. This accessory protein could facilitate MEK/ERK uncoupling to target different substrates. Our results add to the importance of identifying this unknown substrate for MEK. Last, our findings further suggest the reinterpretation of studies that assumed ERK-mediated activities based on MEK inhibition, especially when the upstream signaling includes MLK. Overall, we find that MLK3-MEK signaling is critical for repair.

In contrast to previous results (*9*, *34*), we did not find any changes to repair responses by blocking K^+^ efflux, nor by blocking p38 or JNK1/2. However, the previous studies used different toxins and/or different time points. Notably, LLO was previously reported to trigger MEK downstream of K^+^ efflux (*9*, *34*), which we did not recapitulate with SLO, PFO, or ILY. It is possible that LLO triggers different membrane repair responses compared to SLO (*49*), which could account for the difference. Previous work detecting protective effects of p38 or JNK1/2 activation used longer (hours/days) time points (*50*–*52*), after toxin exposure, in contrast to the rapid (<30 min) repair responses measured here. Microparticle shedding from the plasma membrane occurs after p38 or ERK1/2 activation in other systems (*53*, *54*), suggesting that there are early and late stages of signaling that promote repair. This could account for the finding that cells reseal subsequent wounds faster than initial ones (*55*). However, it remains to be determined whether MEK contributes to patch repair.

Our results reveal that MEK signaling acts in parallel to the well-established ceramide-mediated protection (*14*, *23*, *24*). We modulated ceramide levels without perturbing sphingomyelin levels by blocking glucosylceramide synthesis, so we attribute the protective response to elevated ceramide levels, rather than to loss of sphingomyelin. However, we cannot rule out a decrease in glucosylceramide. Knockout of *UGCG*, which also blocks glucosylceramide synthesis, protected cells from ILY (*40*). This pathway represents a branch of Ca^2+^-dependent repair that acts in parallel to MEK, indicating that Ca^2+^ triggers multiple pathways to promote repair. Since ceramide is a target for A1 (*56*), which is also MEK independent, it is possible that ceramide mediates its protective effects via A1 and/or other annexins to help reseal damage. Thus, annexins may serve as downstream effectors for multiple repair pathways.

We found that annexin membrane recruitment is controlled by different upstream signals. Timely A2, but not A1 or A6, membrane recruitment required MEK. Consistent with previous studies (*4*, *10*, *13*), A2 was recruited to the membrane first. However, MEK inhibition drove faster recruitment of A1 and A6, which we interpret to be a parallel, compensatory mechanism triggered by the cell’s failure to limit intracellular Ca^2+^ flux. Consistent with previous results (*32*, *57*), we found that A1 and A6 increased resistance to CDCs. Similarly, deletion of either A1 or A2 increased cell sensitivity to CDCs. Notably, B6 BMDMs, which have a truncated A6 (*32*), are more susceptible to CDCs than CD-1 BMDMs with full-length A6. While other differences may underlie CD-1 resistance, our work suggests that comparison of A6^−/−^ mice to WT B6 mice may not reveal the impact of A6 on membrane repair. These annexins bind to different lipids. A2 is recruited to caveolar domains (*58*), while A6 binds to phosphatidylserine and cholesterol, and A1 binds to phosphatidylserine and ceramide (*56*, *59*, *60*).We conclude that annexins serve distinct roles in membrane repair and that they are activated by multiple mechanisms to ensure a repair response.

We report the first signaling pathway needed for microvesicle shedding during repair. We found that shedding of A1^+^ and A6^+^ microvesicles was halved by MEK inhibition. This implies that A1 and A6 are markers of shedding but that additional mechanisms are needed to promote robust shedding. A2 recruitment might be needed for full shedding, but we did not detect A2 on shed vesicles. Annexins may act to clog the breach and are incidentally shed along with the rest of the damage, or A2 may be needed to initiate protein-catalyzed shedding. It is also possible that MEK targets A2 along with other factors needed for shedding, such as ESCRT-III recruitment. Alix is one possible target because Alix is a phosphoprotein that recruits ESCRT-III during multivesicular body formation (*61*) and is recruited to the membrane during repair. During membrane repair, ALG-2 may recruit Alix to the plasma membrane after Ca^2+^ influx (*6*). However, after ALG-2 binding, Alix needs to be stabilized by an accessory protein, such as HIV Gag or Tsg101 (*17*), and there is no role for Tsg101 in membrane repair (*6*, *18*). It is possible that that another protein may stabilize Alix. Phosphorylation by MEK could potentially replace or complement Alix stabilization or ALG-2 in repair. Our work opens new avenues of research into the integration of MEK with ESCRT recruitment.

Our findings shed new light on the interplay between annexins and MEK. An autocrine MEK-A2 loop could contribute to faster repair. A2 up-regulation activates the MEK/ERK pathway in tumors, while MEK can enhance A2 expression (*62*, *63*). PKC inhibition did not alter cell survival although PKC phosphorylation of A2 on Ser^25^ inhibits A2 membrane translocation (*11*). Since A2 is also involved in fibrin homeostasis, p53-mediated apoptosis and drug resistance (*64*, *65*), MLK3/MEK/annexin signaling may serve other roles beyond CDC resistance. Future avenues of research opened by our work include determining whether other annexins are involved in MEK-dependent repair and how MEK regulates the phosphorylation of A2.

Our work had limitations due to the experimental system and the sheer breadth of this pathway. While we ruled out PKC and ceramide as MLK3 activators, the presumptive intermediate signaling step between Ca^2+^ influx and MLK3 remains unknown. Similarly, while we identified A2 as one downstream target of MEK, the signaling pathway by which MEK activates A2 remain unknown. We expect the interaction to be indirect, and MEK to target more proteins than A2, although this remains to be shown. We used inhibitors to map this pathway because genetic ablation of signaling intermediates such as MEK is lethal, and broad spectrum inhibitors (e.g., pan-Raf or pan-MLK inhibitors) cover redundancies built into the system that are challenging to individually knockout. However, we did validate the key inhibitor using siRNA. Last, we restricted our analysis of repair to a subset of pathogenically relevant CDCs and did not examine other sources of injury, including laser wounding, mechanical injury, related membrane attack complex-perforin (MAC-PF) proteins, or smaller bacterial toxins. This work builds a framework for future studies to compare repair between different insults.

Overall, this study broadens our understanding of repair responses to CDCs to include MEK. MEK-dependent repair is a critical survival mechanism because it was conserved across all cell types examined and it resisted multiple CDCs. MEK had a reduced impact on ILY, potentially due to ILY targeting CD59 for binding instead of cholesterol. Overall, we found that MEK contributes up to 70% of Ca^2+^-dependent repair. We provide a mechanistic foundation for cardiomyocyte survival during acute cardiotoxicity and SLO-mediated onset of arrhythmia (*2*), in septic cardiomyopathy. This pathway suggests new antimicrobial targets to enhance cellular resistance to pore-forming toxins during NSTIs and septic cardiomyopathy.

## MATERIALS AND METHODS

### Reagents

All reagents were from Thermo Fisher Scientific (Waltham, MA, USA) unless otherwise noted. Inhibitors were reconstituted in DMSO at 20 mM. Inhibitors were incubated with cells for 30 min in serum-free medium at 20 μM before and included during each assay, unless otherwise mentioned. The MEK1/2 inhibitor U0126 was from Cell Signaling Technology (catalog no. 9903S, Danvers, MA, USA) or Tocris (catalog no. 1144, Minneapolis, MN, USA), while trametinib-GSK1120212 (catalog no. S2673) and PD0325901 (catalog no. S1036) were from Selleckchem (Houston, Texas, USA). The MLK inhibitor URMC-099 (catalog no. 19147) was from Cayman Chemical (Ann Arbor, Michigan, USA). The p38 MAPK inhibitor SB203580 (catalog no. 1202) and pan-PKC inhibitor Go6983 (catalog no. 2285) were from Tocris. Go6983 was used at 100 nM. The MAPK9/JNK1/2 inhibitor SP600125 (catalog no. S1460), pan-RAF inhibitor LY3009120 (catalog no. S7842), and PAK inhibitor FRAX597 (catalog no. S7271) were from Selleckchem. PPMP was from Avanti Polar Lipids (Alabaster, AL, USA) (catalog no. 870792). PPMP was used at 40 μM in complete medium and incubated with cells for 48 hours before experiments. The Rho associated coiled-coil containing protein kinase (ROCK) inhibitor Y-27632 dihydrochloride was from Focus Biomolecules (Plymouth Meeting, PA USA) (catalog no. 10-2301) and used at 10 μM for cardiomyocyte differentiation. Specificity and functionality for inhibitors were assessed by titrating them and analyzing them by Western blots and flow cytometry. siRNA for MEK1, MEK2, ERK1, ERK 2, MLK3, A1, and A2 were designed and ordered from Cytiva (Marlborough, MA, USA). Oligonucleotide sequences are available on request. The anti-A1 monoclonal antibody (mAb) (clone: CPTC-ANXA1) and anti–Lamin A/C mAb (clone: MANLAC1(4A7) were deposited to the Developmental Studies Hybridoma Bank (DSHB) by Clinical Proteomics Technologies for Cancer and G.E. Morris, respectively. These antibodies were obtained from the DSHB, created by the National Institute of Child Health and Human Development of the National Institutes of Health (NIH) and maintained at the University of Iowa, Department of Biology, Iowa City, IA, USA. Anti-SLO mAb (clone: 6D11, catalog no. NBP1-05126) was obtained from Novus Biologicals (Littleton, CO, USA). Anti-MEK (91122 l), anti–phospho-MEK-[Ser^217^/Ser^221^] (9121S), anti-MLK3 (2817S), anti-ERK p44/42 (9102S), anti–phospho-ERK[Thr^202^/Tyr^404^] (9101S), anti-p38 (9212S), anti–phospho-p38 (94511S), anti-SAPK/JNK1/2 (9252S), and anti-SAPK/JNK1/2 (9255S) rabbit polyclonal antibodies were from Cell Signaling Technologies. Goat anti-mouse (711-035-151) and anti-rabbit (711-035-152) horseradish peroxidase (HRP)–conjugated antibodies were from Jackson ImmunoResearch (West Grove, PA, USA).

### Plasmids

The pBAD-gIII plasmid encoding His-tagged SLO was a gift from Michael Caparon (Washington University in St. Louis, MO, USA) (*28*). Cysteine-less, His-tagged PFO in pET22 (*66*) and cysteine-less, His-tagged ILY in pTrcHisA (*67*) were gifts from R. Tweten (University of Oklahoma Health Sciences Center, Oklahoma City, OK, USA). Human A6 fused to YFP or cyan fluorescent protein (CFP) and pig A1 fused to YFP or CFP were gifts from A. Draeger (University of Bern, Bern, Switzerland) (*14*). A2-GFP (catalog no. 107196) and A2-GFP lacking all calcium binding sites (catalog no. 107197) (mutA2) were from Addgene (*68*). OlyA fused to mCherry in pET21c(+) was a gift from K. Sepčić (University of Ljubljana, Ljubljana, Slovenia) (*39*). Cysteine-less, His-tagged SLO codon-optimized for *Escherichia coli* expression was synthesized by Genewiz (New Brunswick, NJ) and cloned into pBAD-gIII. QuikChange mutagenesis was used to introduce the E69A mutation in OlyA to remove the cholesterol requirement for OlyA binding to sphingomyelin (*38*) and generate the G395V/G396V monomer-locked mutation (*28*) in codon-optimized SLO. Primer sequences are available upon request.

### Recombinant toxins

Toxins for assays were induced and purified as previously described (*8*, *15*, *69*). Briefly, toxins were induced with 0.2% arabinose (SLO) or 0.2 mM isopropyl-β-d-thiogalactopyranoside (PFO, ILY, or OlyA) for 3 hours at room temperature and then purified using Nickel–nitrilotriacetic acid resin. The protein concentration was determined by Bradford assay. The hemolytic activity of each toxin was determined as previously described (*8*, *15*, *69*) using human red blood cells (Zen-Bio, Research Triangle Park, NC, USA). One hemolytic unit (HU) is defined as the quantity of toxin required to lyse 50% of a 2% human red blood cell solution in 30 min at 37°C in 2 mM CaCl_2_, 10 mM Hepes (pH 7.4), and 0.3% bovine serum albumin in phosphate-buffered saline (PBS) (*8*, *69*). We used HU per milliliter to normalize toxin activities in each experiment and to achieve consistent cytotoxicity across toxin preparations. While protein yields were higher with codon-optimized WT SLO, no difference in specific activity was observed between WT and codon-optimized WT SLO. The nonhemolytic SLO ML had a specific activity of <10 HU/mg and was used at a mass equivalent to active SLO. The highest toxin concentration that killed <20% of target cells was defined as the sublytic dose (*15*). For HeLa cells, the sublytic dose used was 250 HU/ml for SLO, 125 HU/ml for PFO, and 250 HU/ml for ILY. SLO was fluorescently labeled as previously described (*8*). Briefly, purified Cys-less SLO was gel-filtered into 100 mM sodium bicarbonate (pH 8.5) using a Zeba gel filtration column according to the manufacturer’s instructions. Sufficient monoreactive Cy5 dye (Cytiva) to label 0.25 mg of protein was reconstituted in 100 mM sodium bicarbonate, added to the SLO, and incubated for 1 hour at room temperature. Any unconjugated dye was removed by gel filtration of conjugated SLO into PBS. Dithiothreitol (5 mM) was added to the toxin, which was snap-frozen on dry ice.

### Mice

All experimental mice were housed and maintained according to Texas Tech University Animal Care and Use Committee (TTU IACUC) standards, following the Guide for the Care and Use of Laboratory Animals (eighth edition, NRC 2011). TTU IACUC approved animal use. B6 mice or Casp1/11^−/−^ mice on the B6 background were purchased from the Jackson Laboratory (Bar Harbor, ME, USA) (stock nos. 000664 and 016621, respectively) and bred in-house. BM from CD-1 mice euthanized for other purposes were generously provided by the TTU Animal Care Services. BMDMs were prepared using mice of both genders aged 6 to 15 weeks as described below; no gender effects were observed. Mice were euthanized by asphyxiation through the controlled flow of pure CO_2_, followed by cervical dislocation.

### Cell culture

All cell lines used were maintained at 37°C with 5% CO_2_. HeLa cells [ATCC (Manassas, VA, USA) CCL-2] and HEK-293 (ATCC CRL-1573) were cultured in Dulbecco’s modified Eagle’s medium (DMEM; Corning, Corning, NY, USA) supplemented with 10% Equafetal bovine serum (Atlas Biologicals, Fort Collins, CO, USA) and 1× l-glutamine (D10) and 1× penicillin and streptomycin. B6 WT, Casp1/11^−/−^, and CD-1 BMDMs were isolated from BM and cultured as previously described (*15*, *70*). We differentiated BMDMs for 7 to 21 days before experiments in DMEM supplemented with 30% L929 cell supernatant, 20% premium fetal bovine serum (VWR Seradigm, Radnor, PA, USA), 1× l-glutamine, and 1 mM sodium pyruvate. Human (male) iPSC-derived ventricular cardiomyocytes (catalog no. ax2508, Axol Biosciences, Cambridge, UK) were differentiated and cultured for 7 to 10 days per the manufacturer’s instructions. Briefly, 2 to 5 × 10^4^ cells in plating medium [cardiomyocyte maintenance medium (Axol Biosciences) supplemented with 10% premium fetal calf serum and 10 μM ROCK inhibitor (Y-27632)] were plated on 96-well plates or on 35-mm glass-bottom dish (MatTek, Ashland, MA, USA) after coating with a 1:100 dilution of Matrigel (Corning) in RPMI 1640 for 2 hours at 37°C. The next day, the culture medium was replaced with fresh, prewarmed cardiomyocyte maintenance medium, and then half of the volume of the medium was replaced every 2 days with fresh, prewarmed cardiomyocyte maintenance medium.

### Transfection

HeLa cells were plated at 2 × 10^5^ cells per 35-mm glass-bottom dish, 35-mm dish, or per well of a six-well plate and transfected with 500 ng of fluorescent A6 or A1, or 750 ng of A2 or mutA2 GFP using Lipofectamine 2000 in Opti-MEM 2 days before imaging or cytotoxicity assays. The D10 medium was replaced on the day after transfection. Transfection efficiencies for each construct ranged between 65 and 80% for each experiment. For RNAi, HeLa cells were plated at 2 × 10^5^ cells in six-well plates and transfected the following day with 10 nM siRNA using Lipofectamine 2000 in Opti-MEM for 48 to 72 hours.

### Cytotoxicity assays

#### MTT assay

The MTT [3-(4,5-dimethylthiazol-2-yl)-2,5-diphenyltetrazolium bromide] assay was performed and analyzed as described for cardiomyocytes or HeLa cells (*15*). A total of 2 × 10^4^ iPSC-derived cardiomyocytes differentiated in 96-well plates were pretreated with DMSO or 20 μM U0126. After incubation, medium was aspirated and replaced with phenol red–free DMEM with 25 mM Hepes (pH 7.4) and 0.1 mM β-mercaptoethanol (βME). Cardiomyocytes were challenged with SLO (62.5 HU/ml) for 30 min at 37°C. HeLa cells were plated in 96-well plates, pretreated with DMSO or 20 μM U0126, and challenged with SLO (31 to 2000 HU/ml) for 0.5 to 24 hours at 37°C in DMEM. After SLO challenge, cardiomyocytes or HeLa cells were centrifuged at 1200*g* for 5 min at 4°C to remove the medium. Phenol red–free DMEM with 1.1 mM MTT was added, and cells were incubated at 37°C for 4 hours. SDS-HCl was used to dissolve formazan at 37°C overnight, and absorbance was measured at 570 nm using a plate reader (Bio-Tek, Winooski, VT, USA). We calculated the percentage of viable cells as % Viable = (Sample − Background)/(Control − Background) × 100. Then, the percentage of specific lysis was calculated as 100 − % Viable.

#### Flow cytometry assay

Cytotoxicity was performed as described (*8*, *15*). All cells were serum-starved in DMEM for 30 min at 37°C and then pretreated with any inhibitors or DMSO in serum-free medium for 30 min at 37°C. Then, 1 × 10^5^ cells were challenged in suspension with various concentrations of toxins for 30 min at 37°C in RPMI supplemented with 2 mM CaCl_2_ (RC) and propidium iodide (PI; 20 μg/ml) (catalog no. P4170, Sigma-Aldrich, St. Louis, MO, USA), as well as any inhibitors used or DMSO. Cells were analyzed on a four-laser Attune Nxt flow cytometer. Assay variations included changes to the medium (swap 2 mM EGTA in place of 2 mM CaCl_2_) or the viability dye [TO-PRO3 (2 μg/ml), catalog no. T3605]. TO-PRO3 and PI give similar results in the cytotoxicity assay (*15*). For analysis of cell lysis, we gated out the debris and then quantified the percentage of cells with high dye fluorescence (2 to 3 log shift) (dye high), low dye fluorescence (~1 log shift) (dye low), or background dye fluorescence (dye negative). Populations of cells marked “dye negative” and “dye low” remain metabolically active indicating that only the “dye high” populations are dead cells (*5*). We calculated specific lysis as % Specific Lysis = (% Dye High^Experimental^ − % Dye High^Control^) / (100 − % Dye High^Control^) × 100. Transiently permeabilized cells were similarly calculated using the dye low population instead of the dye high populations. The toxin dose needed to kill 50% of cells was defined as the LC_50_ and was determined by regression of the linear portion of the kill curve using Excel (Microsoft, Redmond, WA, USA) as previously described (*15*).

### Live-cell imaging

#### Calcium flux assays

HeLa cells were plated at 2 × 10^5^ cells per 35-mm glass-bottom dish 2 days before imaging. Cells were pretreated with DMSO or U0126 and simultaneously labeled with 4 μM cell-permeant Fluo-4 AM (catalog no. F14201) for 30 min at 37°C. After a brief wash, Fluo-4 acetomethoxy (AM)–labeled cells were challenged with the following sublytic toxin doses: SLO (250 HU/ml), PFO (125 HU/ml), or ILY (250 HU/ml), or a mass equivalent of SLO ML; and imaged at 37°C in RPMI, 25 mM Hepes (pH 7.4), 2 mM CaCl_2_, and TO-PRO3 (2 μg/ml) for 45 min using a Yokogawa CSU-X spinning disc confocal microscope (Intelligent Imaging Innovation, Denver, CO, USA) with a 60×, 1.49 NA (numerical aperture) oil immersion objective and recorded using an Evolve 512 EMCCD camera (Photometrics, Tucson, AZ, USA) at 1 to 3 s per frame. Fluo-4 AM was excited using the 488-nm laser, while TO-PRO3 was excited with the 640-nm laser. After 45 min, the assay was stopped by adding an equal volume of 2% Triton X-100 to give a final concentration of 1%. Triton addition served as a control for TO-PRO3 uptake and dye loss.

The extent of calcium flux in cells was assessed by measuring intensity from the middle of the cell over time by plotting Z profiles in FIJI (ImageJ, NIH, Bethesda, MD, USA). TO-PRO3 uptake was determined in cells by plotting the Z profile of a nuclear subsample over time. We calculated fluorescence intensity over time for individual cells in each toxin group and then normalized each group to their brightest point to measure the temporal changes. We calculated the number of dead cells in each time point per experiment by converting monochromatic blue (TO-PRO3) channel into masks and analyzing particles in FIJI. Experimental cells that expressed at least two-third–maximal fluorescence intensity of TO-PRO3 (after Triton X-100) were considered dead. We categorized cells based on their survival: “died in 5 min,” “died between 5 and 30 min,” and “survived >30 min.” No cells died between 30 min and the end of the experiment.

#### Fluorescent annexin shedding assays

HeLa cells were plated at 2 × 10^5^ cells per 35-mm glass-bottom dish and transfected with 500 ng of A6-YFP or A1-YFP or 750 ng of A2-GFP or mutA2-GFP using Lipofectamine 2000 2 days before imaging. The transfection efficiency was ~65 to 80%. A1- or A6-transfected cells were challenged with a sublytic toxin dose for SLO (250 HU/ml), PFO (125 HU/ml), or ILY (250 HU/ml) or mass equivalent of SLO ML and imaged at 37°C in RPMI, 25 mM Hepes (pH 7.4), and 2 mM CaCl_2_ with TO-PRO3 (2 μg/ml) for 45 min. For A2 and mutA2, the sublytic dose was SLO (500 HU/ml), PFO (250 HU/ml), or 500 HU/ml. A1-YFP– and A6-YFP–positive cells were imaged either as described above for calcium imaging (*15*) or using a Fluoview 3000 confocal microscope (Olympus, Tokyo, Japan) equipped with a 60×, 1.42 NA oil immersion objective and recorded using the resonant scanner at 15 frames/min. A2-GFP– and mutA2-GFP–positive cells were imaged using the Fluoview 3000 confocal microscope. GFP and YFP were excited using a 488-nm laser line, while TO-PRO3 was excited with a 640-nm laser line. The assay was terminated by adding an equal volume of 2% Triton X-100 to give a final concentration of 1% after 45 min. Addition of Triton X-100 served as a control on TO-PRO3 uptake and annexin translocation. Microvesicles released from A6-YFP–, A1-YFP–, or A2-GFP–positive cells were manually counted using every second frame and expressed as microvesicles per number of cells per minutes (*15*). The proportion of cells undergoing fluorescent annexin translocation was calculated by comparing the annexin fluorescent intensity at 15 to 1 min after toxin addition in DMSO-treated cells. Cells with 80% reduction in fluorescence intensity of the initial value were considered to show annexin translocation (*15*). The extent of fluorescent annexin depletion from cells was measured by calculating intensity from the central region of the cell over time by plotting “Z” profiles in FIJI in the translocated cells. Similarly, TO-PRO3 uptake was determined by plotting the Z profile of a nuclear subsample over time, as previously described (*15*). For A1-YFP, A6-YFP, A2-GFP, mutA2-GFP, and TO-PRO3, the data were normalized to the integrated intensity of the brightest point. The *t*_1/2_ for annexin depletion, membrane accumulation, or TO-PRO3 uptake was calculated by measuring the half-maximal intensity between the starting intensity and the average intensity of the last 4 min before Triton X-100 addition. Membrane accumulation of annexins (A1, A6, A2, or mutA2) over time was calculated by determining the changes in the fluorescence intensity along the edges of the cells of translocated cells by plotting the Z profile in FIJI. The intensity was then normalized on a per-cell basis, and individually analyzed cells were plotted. From the multiplane images, several cells (>25) were analyzed from at least three independent experiments and graphed using Microsoft Excel. Quantified values for individual cells were represented with GraphPad.

#### Cardiomyocyte beating and cell death assay

A total of 5 × 10^4^ iPSC-derived cardiomyocytes on 35-mm glass-bottom dishes were pretreated with DMSO or 20 μM U0126 and Cell Mask Green (1:4000) at 37°C for 30 min. After incubation, medium was aspirated and replaced with phenol red–free DMEM with 25 mM Hepes (pH 7.4), 0.1 mM βME, and TO-PRO3 and then challenged with SLO (62.5 HU/ml). Differential interference contrast (DIC) images were collected for 1 min before toxin addition for baseline. Confocal and DIC images of cells were collected for ~45 min at 37°C after toxin challenge. Cell contractions per minute were expressed as beats per minute measured in ImageJ using MUSCLEMOTION macros (*71*).

#### Bleach correction and supplementary movies

Images were exported as .tif and split into individual monochromatic red, green, and blue channels. For display (but not analysis), bleach correction was performed as previously described (*15*) on the green channel cells by histogram matching using FIJI, followed by a median pass filter. The monochromatic images were then merged to form RGB .tif files, time-stamped, annotated, and exported as AVIs for supplementary movies.

#### Isolation of microvesicles

Microvesicles were isolated as previously described (*5*, *8*). A total of 5 to 10 × 10^6^ cells were harvested, resuspended in RC at 2.5 × 10^6^ to 5 × 10^6^ cells/ml, challenged with a sublytic dose of toxin, and incubated for 15 min at 37°C. Challenged cells were then centrifuged at 2000*g* for 5 min, solubilized at 95°C in SDS-sample buffer for 5 min, and sonicated. Supernatants were spun at 100,000*g* in a Beckman Coulter Optima XPN-80 ultracentrifuge using a Beckman SW 41 Ti rotor for 40 min at 4°C. The microvesicle pellet was solubilized at 95°C in 4× SDS-sample buffer. We have previously demonstrated (*5*, *72*) that vesicles triggered by CDCs best fit the definition of microvesicle. These microvesicles are nontoxic to cells and do not fuse with the plasma membrane of cells that internalize these microvesicles (*72*).

#### SDS-polyacrylamide gel electrophoresis and immunoblotting

Samples were resolved on 10% polyacrylamide gels at 160 V for 90 min and transferred to nitrocellulose in an ice bath with transfer buffer (15.6 mM tris and 120 mM glycine) at 300 mA for 60 min. Blots were blocked using 5% skim milk in 10 mM tris-HCl, 150 mM NaCl, and 0.1% Tween 20 (pH 7.5). Portions of the blot were incubated with one of the following primary antibodies for 2 hours at room temperature: CPTC-A1-3 anti-A1 (1:250) mAb, anti-A2, AC-15 anti–β-actin (1:5000) mAb, or rabbit polyclonal antibodies: anti-MEK, anti–phospho-MEK [Ser^217^/^221^], anti-MLK3, anti–phospho-MLK3 [Thr^227^, Ser^281^], anti-ERK p44/42, anti–phospho-ERK [Thr^202^/Tyr^404^], anti-p38, anti–phospho-p38, anti-SAPK/JNK1/2, and anti–phospho-SAPK/JNK1/2 each at 1:1000 dilution. Next, blots were incubated with HRP-conjugated anti-mouse or anti-rabbit immunoglobulin G antibodies (1:10,000) and developed with enhanced chemiluminesence (ECL): 0.01% H_2_O_2_ (Walmart, Fayetteville, AR, USA), 0.2 mM p-Coumaric acid (Sigma-Aldrich), 1.25 mM luminol (Sigma-Aldrich) in 0.1 M tris (pH 8.4), ECL Plus Western Blotting Substrate (catalog no. 32134), or ECL Prime Western Blotting Reagent (catalog no. RPN2232, Cytiva).

EM rapid-freeze, deep-etch microscopy was performed as previously described (*5*). HeLa cells were seeded onto coverslips, pretreated with 20 μM U0126 or its inactive analog U0124 for 30 min, challenged with a sublytic dose of SLO for 5 min at 37°C, washed, fixed in 2% glutaraldehyde, and analyzed by rapid-freeze, deep-etch EM.

### Statistical analysis

Prism 8.1 (San Diego, CA, USA) or Excel was used for statistical analysis. Data are represented as means ± SEM as indicated. The LC_50_ for toxins was calculated by linear regression using the linear portion of the death curve. Significance between curves was determined by logistic regression and comparing parameters. Statistical significance was determined by one-way analysis of variance (ANOVA) or repeated-measures ANOVA; *P* < 0.05 was considered to be statistically significant. Graphs were generated in Excel or Prism.

## Notes

View/request a protocol for this paper from *Bio-protocol*.
