# Supplementary material for: Membrane repair triggered by cholesterol-dependent cytolysins is activated by mixed lineage kinases and MEK
Source: Sci Adv. 2022 Mar 16;8(11):eabl6367. doi: 10.1126/sciadv.abl6367 (PMC8926344; doi:10.1126/sciadv.abl6367)
Supplement: Supplementary file 1 — Figs. S1 to S5 [file sciadv.abl6367_sm.pdf]

Science Advances
AAAS

Supplementary Materials for

Membrane repair triggered by cholesterol-dependent cytolysins is activated by mixed lineage kinases and MEK

Sucharit Ray, Robyn Roth, Peter A. Keyel*

*Corresponding author. Email: peter.keyel@ttu.edu

Published 16 March 2022, *Sci. Adv.* **8**, eabl6367 (2022)
DOI: 10.1126/sciadv.abl6367

The PDF file includes:

Figs. S1 to S5
Legends for movies S1 to S10

Other Supplementary Material for this manuscript includes the following:

Movies S1 to S10

FIGURE S1

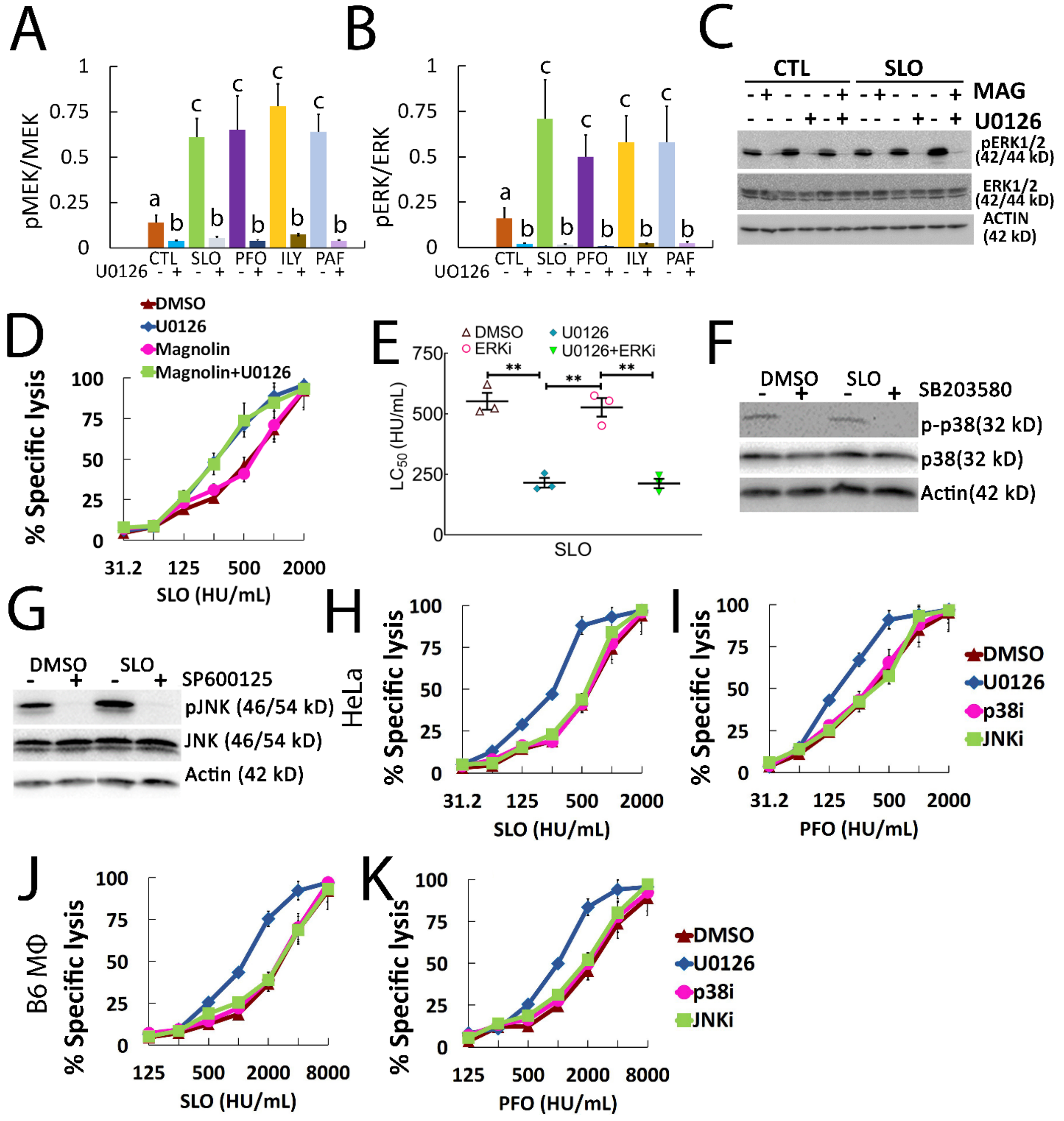

Supplementary Figure S1. Repair responses are independent of ERK, p38 and JNK1/2, related to Fig 1. (A-I) HeLa cells or (J, K) B6 BMDM were pretreated with DMSO, (C-E) 20 µM Magnolin (ERK inhibitor), and/or (A-E, H-K) U0126, (F, H-K) 20 µM SB203580 (p38i), or (G-K) 20 µM SP600125 (JNKi) for 30 min and challenged with (A, B, E, F) 250 HU/mL SLO, or the indicated concentrations of (C, D, G, I) SLO or (H, J) PFO, and analyzed by (A-C, F, G) western blot or (D, E, H-K) flow cytometry. Portions of blots were probed for the indicated proteins followed by HRP conjugated secondary antibodies. Graphs show mean ±S.E.M. of at

least (A, B) four, (D, E) three or nine (H-K) independent experiments. The LC_{50} was calculated as described in the methods. The quantitation in (A, B) is based on the blots used for Fig 1C. The blots show one representative blot from at least three (C) or five (F, G) independent experiments. ** $p<0.01$ Letters (a-c) denote statistically significant ($p < 0.05$) groups for each challenge using repeated-measures ANOVA between groups.

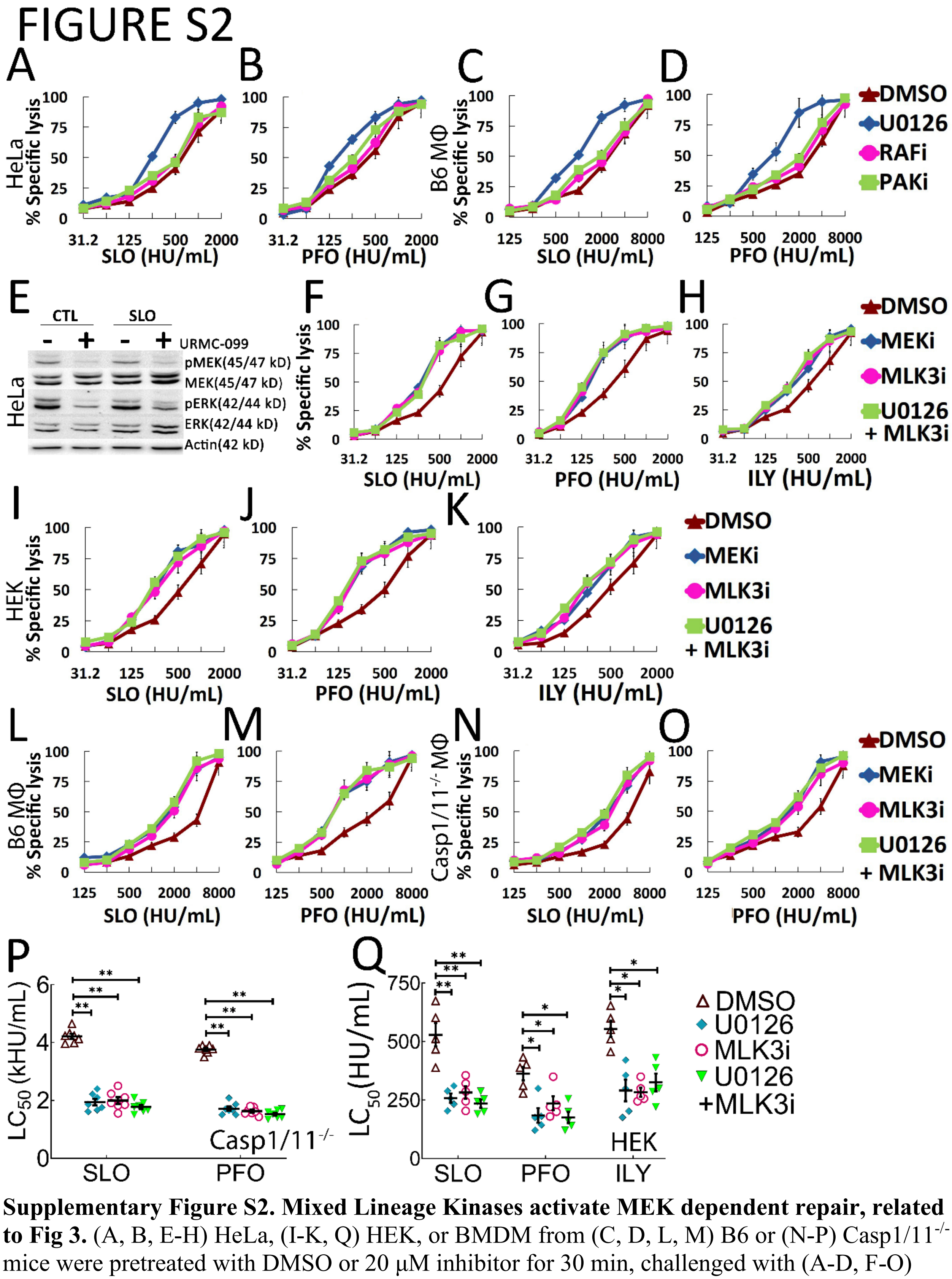

Supplementary Figure S2. Mixed Lineage Kinases activate MEK dependent repair, related to Fig 3. (A, B, E-H) HeLa, (I-K, Q) HEK, or BMDM from (C, D, L, M) B6 or (N-P) Casp1/11$^{-/-}$ mice were pretreated with DMSO or 20 µM inhibitor for 30 min, challenged with (A-D, F-O) SLO, PFO or ILY at the indicated concentrations or (E) 250 HU/mL SLO for 30 min at 37 °C,

and (A-D, F-O) PI uptake analyzed by flow cytometry or (E) phosphorylation analyzed by western blot. Portions of the blot were probed with anti-phospho-MEK, anti-MEK, anti-phospho-ERK, anti-ERK or anti-β-actin followed by HRP conjugated secondary antibodies. (P, Q) The LC_{50} for (I-O) was calculated as described in the methods. PAKi is FRAX597. RAFi is LY3009120. MLK3i is URMC-099. MEKi is U0126. Data points represent individual experiments. All graphs display the mean ±S.E.M. of at least seven independent experiments. The blot shows one representative experiment from three independent experiments. $*p < 0.05$, $** p < 0.01$

FIGURE S3

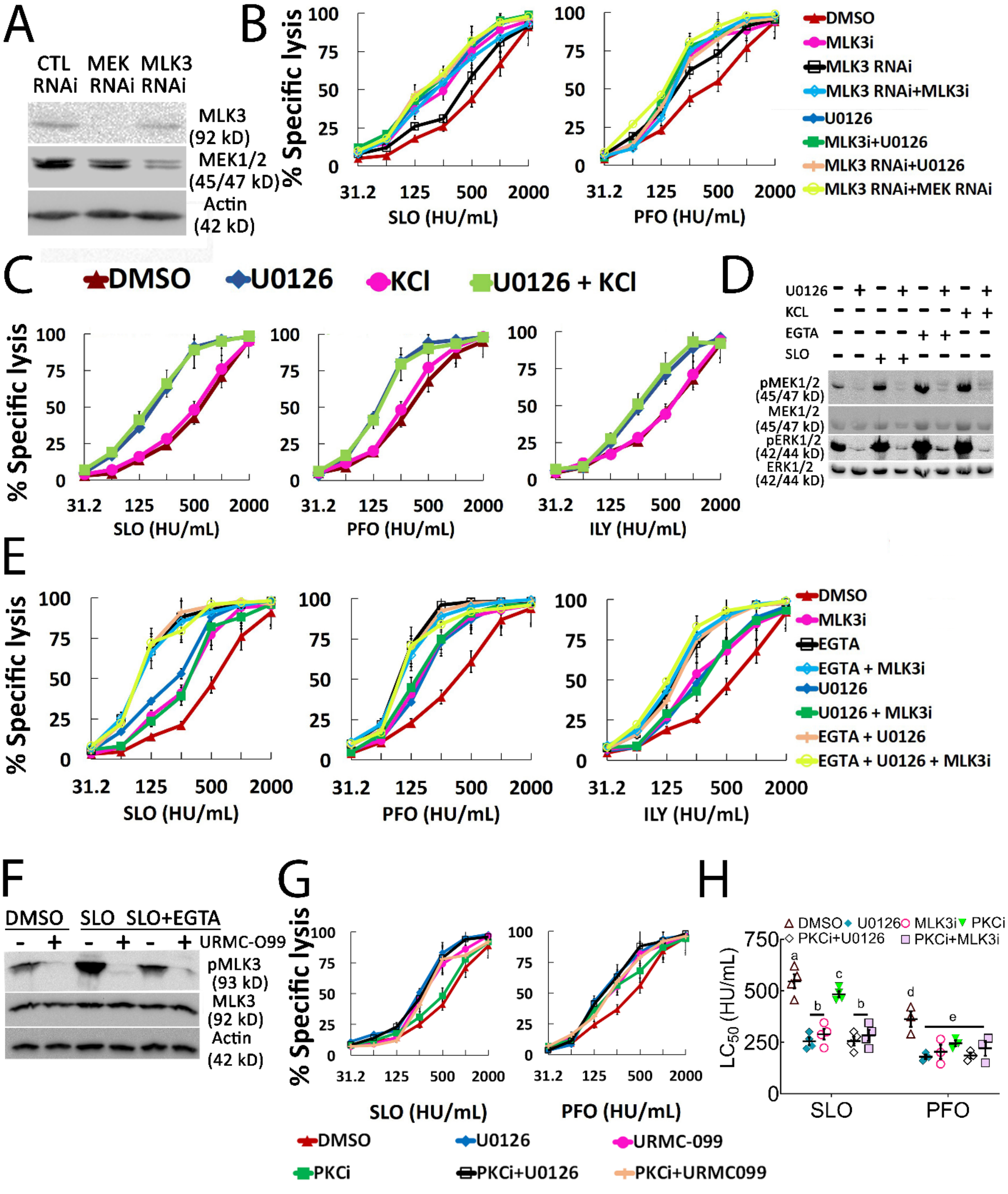

Supplementary Figure S3. MLK3/MEK dependent repair acts downstream of Ca^{2+} influx but is independent of K^{+} efflux, related to Fig 3. (A, B) HeLa cells were treated with control, MEK1/2, ERK1/2, or MLK3 siRNA for 72 h and analyzed by (A) western blot or (B) treated with DMSO or 20 μM inhibitor, then challenged with SLO or PFO for 30 min at 37 °C, and PI

uptake analyzed by flow cytometry. (C, E, G, H) HeLa cells were pretreated with DMSO, 20 μM U0126, 20 μM URMC-099 (MLK3i), or 100 nM Go6983 (PKCi) for 30 min, challenged with SLO, PFO or ILY in the presence of 2 mM $CaCl_2$, 2 mM EGTA or 150 mM KCl and analyzed by (C, E, G, H) flow cytometry. (D) HeLa cells were pretreated with DMSO or 20 μM U0126, challenged with 250 HU/mL SLO, 2 mM EGTA or 150 mM KCl and analyzed by western blot. (F) HeLa cells were pretreated with DMSO, or 20 μM URMC-099 (MLK3i) and challenged with SLO in the presence of 2 mM $CaCl_2$ or 2 mM EGTA and analyzed by western blot. Portions of blots were incubated with anti-phospho-MEK, anti- MEK, anti-phospho-ERK, anti-ERK, anti-phospho-MLK3, anti-MLK3, or anti-β-actin followed by HRP conjugated secondary antibodies. (H) LC_{50} for (G) was calculated as described in the methods. Data points represent individual experiments. All graphs display the mean ±S.E.M. of at least three independent experiments. Blots show one representative experiment from at least three independent experiments. Letters (a-e) denote statistically significant ($p < 0.05$) groups for each CDC using repeated-measures ANOVA between groups.

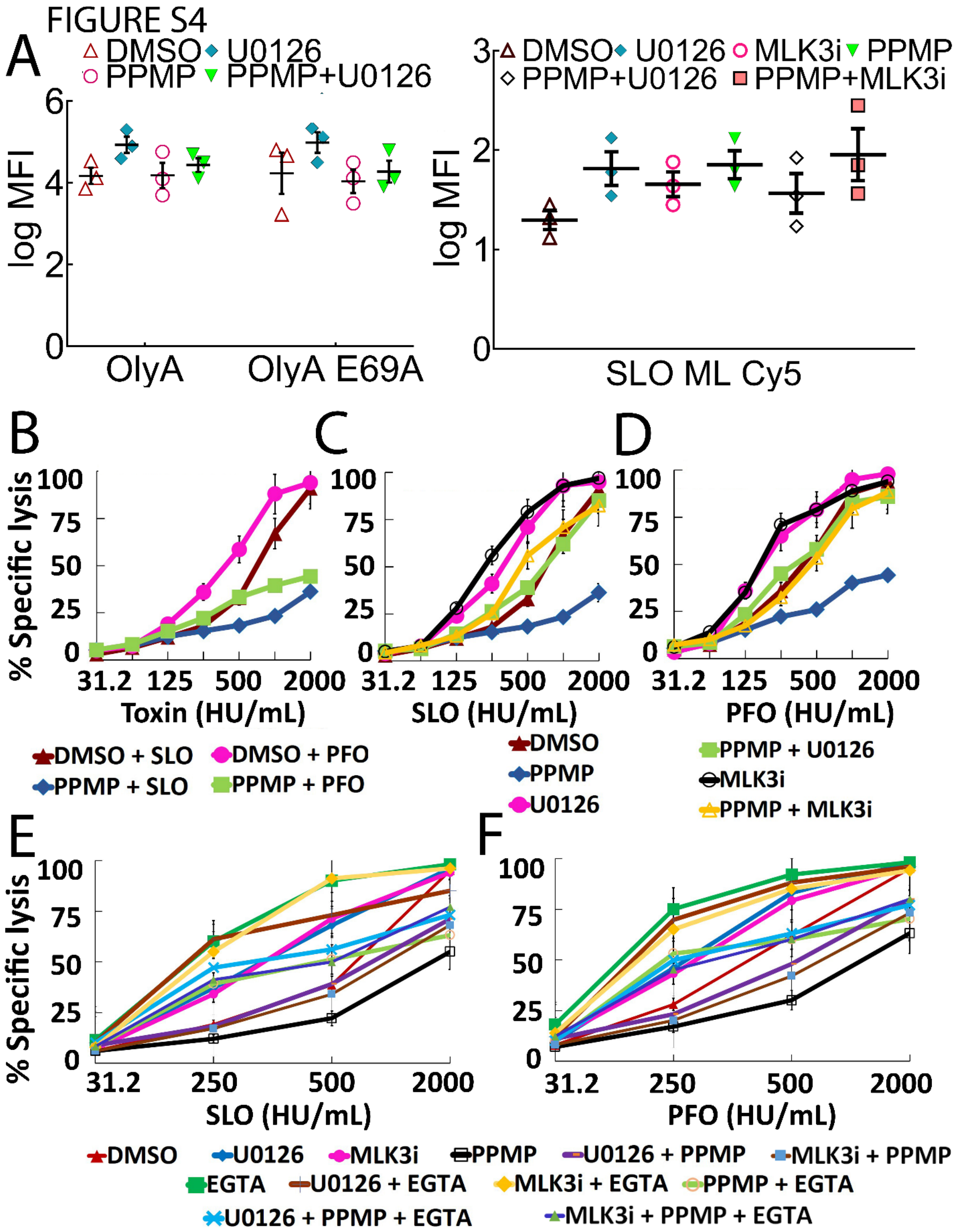

Supplementary Figure S4. Ceramide may activate a parallel repair pathway, related to Fig 4. HeLa cells were pretreated with 40 μM PPMP for 48 h, then DMSO or 20 μM inhibitor for 30

min. Next, cells were (A) labeled with 20 µg/mL of OlyA-mCherry WT or OlyA-mCherry E69A for 5 min or labeled with 100 µg/mL of Cy5-conjugated SLO ML for 30 min, or (B-F) challenged with SLO or PFO for 30 min at 37 °C and toxin binding or PI uptake analyzed by flow cytometry. 2 mM EGTA was used instead of 2 mM $CaCl_2$ where indicated. MLK3i is URMC-099. Data points represent individual experiments. Graphs show the mean ±S.E.M. of at least three independent experiments.

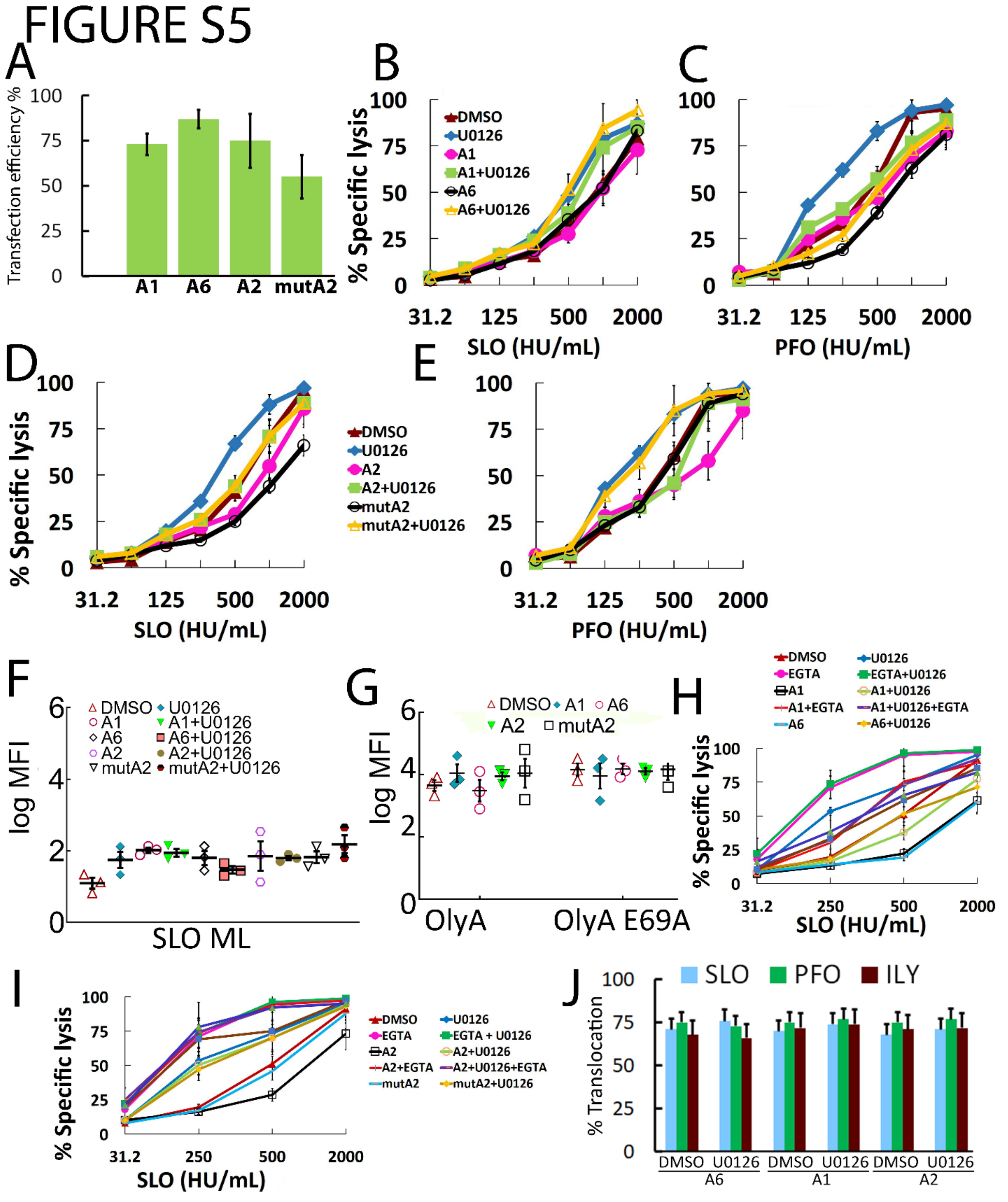

Supplementary Figure S5. Annexins promote survival, related to Fig 7. HeLa cells were untransfected or transfected with A1-YFP, A6-YFP or A2-GFP or mutant A2-GFP (mutA2) for 48 h. (A) The transfection efficiency is shown. (B-F, H, I) Transfected cells were pretreated with DMSO or 20 μM U0126 for 30 min, challenged with (B, D, H, I) SLO or (C, E) PFO for 30 min

and PI uptake analyzed by flow cytometry. 2 mM EGTA was used instead of 2 mM $CaCl_2$ where indicated. (F, G) Transfected cells were labeled with (F) 100 µg/mLCy5 conjugated SLO ML for 30 min or (G) with 20 µg/mL of OlyA-mCherry or OlyA-mCherry E69A for 5 min at 37 °C and toxin binding analyzed by flow cytometry for PI uptake. (J) The fraction of transfected cells which had annexin translocation to the membrane after toxin challenge were measured. Data points represent individual experiments. Graphs show mean ±S.E.M. of at least three independent experiments.

Supplemental Video V1. Cardiomyocytes are sensitized to SLO after MEK inhibition, related to Fig 1. Human cardiomyocytes were incubated with DMSO (left panel) or 20 μM U0126 (right panel) and Cell Mask Green (green) for 30 min. Media was replaced with DMEM, 25 mM HEPES, pH 7.4, βME and TO-PRO3 (blue). Cardiomyocytes were challenged with 62.5 HU/mL of SLO. (A, B) DIC images 1 min before toxin addition show baseline. (C, D) Confocal imaging of cells for ~45 min at 37°C after toxin challenge. DIC: 2 frame/sec, Confocal ~ 0.7 frame/sec. Time following toxin addition is shown in min. Scale bar = 10 μm.

Supplemental Video V2. MEK prevents rapid Ca^{2+} influx from SLO, related to Fig 5. HeLa cells were incubated with Fluo-4 and vehicle DMSO (left panel) or 20 μM U0126 (right panel) for 30 min in RPMI. The media was replaced with imaging buffer, including TO-PRO-3 (blue). Cells were imaged by confocal microscopy at 1-3 frames/sec for ~45 min at 37°C. Sublytic (A) SLO or (B) SLO ML were added 5 min after imaging started. Triton-X-100 was added at the end as a positive control for cell permeabilization. Images were bleach-corrected by histogram matching. Time shows min. Scale bar = 10 μm.

Supplemental Video V3. MEK prevents rapid Ca^{2+} influx from PFO and ILY, related to Fig 5. HeLa cells were incubated with Fluo-4 and vehicle DMSO (left panel) or 20 μM U0126 (right panel) for 30 min in RPMI. The media was replaced with imaging buffer, including TO-PRO-3 (blue). Cells were imaged by confocal microscopy at 1-3 frames/sec for ~45 min at 37°C. Sublytic (A) PFO or (B) ILY were added 5 min after imaging started. Triton-X-100 was added at the end as a positive control for cell permeabilization. Images were bleach-corrected by histogram matching. Time shows min. Scale bar = 10 μm.

Supplemental Video V4. MEK drives shedding of A1+ microvesicle during repair from SLO, related to Figs 8 and 9. HeLa cells transfected with A1-YFP (green) were pretreated with DMSO (left panel) or 20 μM U0126 (right panel) for 30 min, challenged with sublytic (A) SLO or (B) SLO ML in the presence of 2 μg/mL TO-PRO3 (blue), and imaged at 37°C by live cell confocal imaging at 1-3.5 frame/second. Triton-X-100 was added at the end as a positive control for cell permeabilization. Images were bleach-corrected by histogram matching. Time shows min. Arrows show regions of microvesicle shedding. Scale bar = 10 μm.

Supplemental Video V5. MEK drives shedding of A1+ microvesicle during repair from PFO and ILY, related to Figs 8 and 9. HeLa cells transfected with A1-YFP (green) were pretreated with DMSO (left panel) or 20 μM U0126 (right panel) for 30 min, challenged with sublytic (A) PFO or (B) ILY in the presence of 2 μg/mL TO-PRO3 (blue), and imaged at 37°C by live cell confocal imaging at 1-3.5 frame/second. Triton-X-100 was added at the end as a positive control for cell permeabilization. Images were bleach-corrected by histogram matching. Time shows min. Arrows show regions of microvesicle shedding. Scale bar = 10 μm.

Supplemental Video V6. MEK drives shedding of A6+ microvesicle during repair, related to Figs 8 and 9. HeLa cells transfected with ANXA6-YFP (green) were pretreated with DMSO (left panel) or 20 μM U0126 (right panel) for 30 min, challenged with sublytic (A) SLO, (B) PFO, (C) ILY, or (D) SLO ML in the presence of 2 μg/mL TO-PRO3 (blue), and imaged at 37°C by live cell confocal imaging at 1.2-3.5 frame/second. Triton-X-100 was added at the end as a

positive control for cell permeabilization. Images were bleach-corrected by histogram matching. Time shows min. Arrows show regions of microvesicle shedding. Scale bar = 10 μm.

Supplemental Video V7. MEK recruits A2 to the site of damage from SLO, related to Figs 8 and 9. HeLa cells transfected with ANXA2-GFP (green) were pretreated with DMSO (left panel) or 20 μM U0126 (right panel) for 30 min, challenged with (A) 500 HU/mL SLO or (B) a mass equivalent SLO ML in the presence of 2 μg/mL TO-PRO3 (blue), and imaged at 37°C by live cell confocal imaging at 1.2-3.5 frame/second. Triton-X-100 was added at the end as a positive control for cell permeabilization. Images were bleach-corrected by histogram matching. Time shows min. Scale bar = 10 μm.

Supplemental Video V8. MEK recruits A2 to the site of damage from PFO and ILY, related to Figs 8 and 9. HeLa cells transfected with ANXA2-GFP (green) were pretreated with DMSO (left panel) or 20 μM U0126 (right panel) for 30 min, challenged with (A) 250 HU/mL PFO or (B) 500 HU/mL ILY in the presence of 2 μg/mL TO-PRO3 (blue), and imaged at 37°C by live cell confocal imaging at 1.2-3.5 frame/second. Triton-X-100 was added at the end as a positive control for cell permeabilization. Images were bleach-corrected by histogram matching. Time shows min. Scale bar = 10 μm.

Supplemental Video V9. A2 requires calcium binding to translocate to the membrane following SLO challenge, related to Figs 8 and 9. HeLa cells transfected with mutA2-GFP (green) were pretreated with DMSO (left panel) or 20 μM U0126 (right panel) for 30 min, challenged with (A) 500 HU/mL SLO or (B) mass-equivalent SLO ML in the presence of 2 μg/mL TO-PRO3 (blue), and imaged at 37 °C by live cell confocal imaging at 1.2-3.5 frame/second. Triton-X-100 was added at the end as a positive control for cell permeabilization. Images were bleach-corrected by histogram matching. Time shows min. Scale bar = 10 μm.

Supplemental Video V10. A2 requires calcium binding to translocate to the membrane following PFO or ILY challenge, related to Figs 8 and 9. HeLa cells transfected with mutA2-GFP (green) were pretreated with DMSO (left panel) or 20 μM U0126 (right panel) for 30 min, challenged with (A) 250 HU/mL PFO or (B) 500 HU/mL ILY in the presence of 2 μg/mL TO-PRO3 (blue), and imaged at 37 °C by live cell confocal imaging at 1.2-3.5 frame/second. Triton-X-100 was added at the end as a positive control for cell permeabilization. Images were bleach-corrected by histogram matching. Time shows min. Scale bar = 10 μm.
